# Genome-wide analysis of WOX genes in upland cotton and their expression pattern under different stresses

**DOI:** 10.1186/s12870-017-1065-8

**Published:** 2017-07-06

**Authors:** Zhaoen Yang, Qian Gong, Wenqiang Qin, Zuoren Yang, Yuan Cheng, Lili Lu, Xiaoyang Ge, Chaojun Zhang, Zhixia Wu, Fuguang Li

**Affiliations:** 1 Xinjiang Research Base, State Key Laboratory of Cotton Biology, Xinjiang Agricultural University, Urumqi, 830052 China; 2 Institute of Cotton Research, Chinese Academy of Agricultural Sciences, Anyang, 455000 China

**Keywords:** *Gossypium hirsutum*, WUSCHEL-related homeobox, Segmental duplication, Transcript factor, Embryogenesis

## Abstract

**Background:**

WUSCHEL-related homeobox (WOX) family members play significant roles in plant growth and development, such as in embryo patterning, stem-cell maintenance, and lateral organ formation. The recently published cotton genome sequences allow us to perform comprehensive genome-wide analysis and characterization of WOX genes in cotton.

**Results:**

In this study, we identified 21, 20, and 38 WOX genes in *Gossypium arboreum* (2n = 26, A_2_), *G. raimondii* (2n = 26, D_5_), and *G. hirsutum* (2n = 4x = 52, (AD)_t_), respectively. Sequence logos showed that homeobox domains were significantly conserved among the WOX genes in cotton, *Arabidopsis*, and rice. A total of 168 genes from three typical monocots and six dicots were naturally divided into three clades, which were further classified into nine sub-clades. A good collinearity was observed in the synteny analysis of the orthologs from At and Dt (t represents tetraploid) sub-genomes. Whole genome duplication (WGD) and segmental duplication within At and Dt sub-genomes played significant roles in the expansion of WOX genes, and segmental duplication mainly generated the WUS clade. Copia and Gypsy were the two major types of transposable elements distributed upstream or downstream of WOX genes. Furthermore, through comparison, we found that the exon/intron pattern was highly conserved between *Arabidopsis* and cotton, and the homeobox domain loci were also conserved between them. In addition, the expression pattern in different tissues indicated that the duplicated genes in cotton might have acquired new functions as a result of sub-functionalization or neo-functionalization. The expression pattern of WOX genes under different stress treatments showed that the different genes were induced by different stresses.

**Conclusion:**

In present work, WOX genes, classified into three clades, were identified in the upland cotton genome. Whole genome and segmental duplication were determined to be the two major impetuses for the expansion of gene numbers during the evolution. Moreover, the expression patterns suggested that the duplicated genes might have experienced a functional divergence. Together, these results shed light on the evolution of the WOX gene family, and would be helpful in future research.

**Electronic supplementary material:**

The online version of this article (doi:10.1186/s12870-017-1065-8) contains supplementary material, which is available to authorized users.

## Background

WUSCHEL-related homeobox (WOX), one of the sub-clades of homeodomain (HD) superfamily, is a plant-specific homeobox (HB) transcription factor, characterized by a short stretch (60–66 residues) of amino acids that form a DNA-binding domain named as homeodomain [1]. Previous reports have shown that WOXs assay a wide variety of important roles in development and growth process of plants, such as in embryonic patterning, stem cell maintenance, and organ formation [2, 3]. In *Arabidopsis thaliana*, which is a model plant, the evolution and function of WOXs have been well studied and characterized. As of date, 16 WOXs have been discovered by whole genome identification [4]; these have been clustered into three clades, namely, the ancient, WUS, and intermediate clades, by phylogenetic analysis [5]. WOXs of lower plants, such as those of green algae and non-vascular moss, *Physcomitrella patens*, belong only to the ancient clade, whereas those of higher plants, such as *Arabidopsis*, sorghum, maize, and rice, are present in all the three clades [1, 2]. *AtWUS* expressed in shoot apical meristem, ovule, and anther has been proven to play important roles in stem cell maintenance [6]. Ectopic expression of *AtWUS* in upland cotton was demonstrated to promote callus dedifferentiation resulting in the formation of embryo callus [7]. *AtWOX3*, as well as its orthologs in maize, *NS1*(*narrow sheath1*) and *NS2*(*narrow sheath2*), perform a highly conserved function during the recruitment of founder cells to form lateral domains of vegetative and floral organs [8]. *AtWOX1* was demonstrated to express in the initiating vascular primordium of the cotyledons during heart and torpedo stages, and its overexpression resulted in defects in the development of meristem and dwarf phenotype [9]. *AtWOX4* was determined to be an essential regulator in auxin-dependent cambium stimulation that regulates lateral plant growth [10]. *AtWOX5*, induced by turanose and auxin, was shown to play vital roles in a correct pattern of root-formation through mediation of auxin homeostasis and by maximizing the auxin content in a restricted area [11]. *AtWOX6/PSF2* was revealed to play important roles in ovule development via the regulation of cell proliferation of the maternal integuments and through differentiation of the megaspore mother cell [12]. *WOX6/HOS9* was also shown to be an essential component, mediating cold tolerance in *Arabidopsis* through a CBF-independent pathway [13]. *AtWOX2* and *AtWOX8* were demonstrated to be essential for determination of the boundary between the cotyledons through the activation of three *CUPSHAPED COTYLEDON* (*CUC*) genes [14]. *STIMPY*/*AtWOX9* was found to be crucial for the growth of vegetative shoot apical meristem through maintenance of cell division and prevention of premature differentiation [15]. Also, *STIMPY*/*AtWOX9* determined the fate of meristem by activation of the cytokinin signaling pathway in meristematic tissue [16]. *WOX11*, being a key regulator of shoot-borne crown development, was associated with the activation of crown root emergence and growth via directly repressing *PR2* to regulate cell proliferation during crown root development [17]. *AtWOX11* directly responds to an auxin maximum, induced by wounding in and around the procambium, and like *AtWOX12*, it positively upregulates *LATERAL ORGAN BOUNDARIES DOMAIN 16* and *29* genes, resulting in the initiation of a leaf procambium or the transition of its neighboring parenchyma cells to root founder cells [18]. *AtWOX13* mainly expresses in meristematic tissues including replum and promotes replum during the development of the *Arabidopsis thaliana* fruits [19]. *AtWOX14* and *WOX4*, regulatory elements downstream of *PHLOEM INTERCALATED WITH XYLEM* (*PXY*), regulate vascular cell division instead of vascular organization, playing crucial roles in stem formation [20].

Cotton is an important fiber crop, which provides the natural renew fiber for textile industry [21]. The roles of WOXs have been well-documented in embryogenesis in *Arabidopsis*; however, the functions of WOXs in cotton, especially in somatic embryogenesis, are largely unknown, thus far. The completion of genome sequencing in cotton allows comprehensive identification and analysis of WOXs in cotton [22–26]. We, therefore, conducted a thorough investigation on WOXs in cotton; the study included the identification of gene families, phylogenetic tree analysis, as well as the analyses of segmental duplication, gene structure, chromosome location, and expression pattern.

## Methods

### Sequence identification

The complete genome sequence data of three cotton species, *Gossypium arboreum* (BJI, version 1.0), *G. raimondii*, (JGI, version 2.0), and *G. hirsutum* (NAU, version 1.1, BJI, version 1.0), available from COTTONGEN (http://www.cottongen.org) [27] were used. The rice (version 7.0), sorghum (version 2.1), cacao (version 1.1), poplar (version 1.1), and maize (version 1.1) genome sequence data were retrieved from JGI (https://phytozome.jgi.doe.gov/pz/portal.html). The amino acid sequences of WOXs from *Arabidopsis thaliana* were acquired from TAIR 10 (http://www.arabidopsis.org); these were used as query sequences to search the *G. arboreum* protein database for candidate sequences employing blastp program. Thereafter, Interproscan 56.0 [28] was used to search for the HB domain (IPR001356) in the candidate sequences, and eventually the WOX sequences were identified. WOXs in rice, sorghum, cacao, *G. hirsutum*, and *G. raimondii* were identified using the same method as employed for *G. arboreum*.

### Conserved sequence and phylogenetic analysis

Multiple sequence alignment was performed using Clustal X 2.0. For sequence logo analysis, the conserved HB domain sequences of WOXs from rice, *Arabidopsis*, and upland cotton were aligned, and the multiple alignment result was submitted to an online tool, WEBLOG [29], for generating the logos. For phylogenetic analysis, the full-length WOX sequences from *Arabidopsis*, rice, sorghum, cacao, *G. arboreum*, *G. raimondii*, and *G. hirsutum* were aligned, and MEGA 7.0 [30] was used to construct a neighbor-joining (NJ) tree. Bootstrap method was used to test the tree with 1000 replicates. Substitution was evaluated by Poisson model using the default parameters. To validate the phylogenetic tree, constructed using the NJ method, the minimum-evolution method was also used. The bootstrap method was used to test the tree with 1000 replicates.

### Chromosome location and collinearity analysis

The gene loci of WOXs were extracted from the annotation gff3-file. Mapchart was then used to obtain the chromosome location [31]. All the protein sequences of upland cotton were included in a local database using Basic Local Alignment Search Tool (BLAST). The entire protein sequences were used as queries to search the above-mentioned database with an e-value of 1e-5. The blastp result was analyzed by MCSCAN to produce the collinearity blocks across the whole genome. The collinearity pairs belonging to WOX family were extracted to draw a collinearity map within WOXs by CIRCOS software [32].

### Calculation of Ka/Ks values

The amino acid sequences from segmentally duplicated pairs and orthologous pairs were first aligned using Clustal X 2.0; thereafter, the aligned sequences were converted to the original cDNA sequences using the PAL2NAL program [33] (http://www.bork.embl.de/pal2nal/). The CODEML program of the PAML package [34] was used to estimate the synonymous (Ks) and nonsynonymous (Ka) substitution rates.

### Annotation and analysis of transposable elements

The *de novo* prediction and homolog search method based on Repbase [35] were used in the present study to identify the repeat content. For the *de novo* analysis, PILER-DF, RepeatModeler, and LTR_FINDER [36, 37] were used to predict the transposable elements (TEs) in the genome. For the analysis using the homology-based approach, the known TE library was used; the TEs were identified at the DNA level with RepeatMasker [38] using Repbase TE. To analyze the function of TEs in the expansion of the WOX family, we identified the TEs located 10,000 and 2,000 bp upstream and downstream of the gene and made statistics of the different types of TEs (mutator-like transposable element (MULE), hAT, CACTA, helitron, retrogenes, and retrotransposons) present.

### Gene structure analysis

*Arabidopsis* and *G. hirsutum* sequences were aligned with Clustal ✕ 2.0, and MEGA 7.0 [30] was used to construct an NJ tree using the method and parameters as described above. The exon positions were acquired from the bed-file, and they were displayed by an online tool, GSDS 2.0 [39].

### Transcriptome data analysis and gene expression heatmap

The raw data of RNA-seq was downloaded from the NCBI Sequence Read Archive (SRA: PRJNA248163). Tophat and cufflinks were used to analyze the RNA-seq expression, and the gene expressions were uniformed in fragments per kilobase million (FPKM) [40]. The expression of WOXs was extracted from the total expression data. Heatmap was drawn by Genesis software [41].

### Real-time PCR

Cotton seeds of TM-1 were obtained from the Institute of Cotton Research of the Chinese Academy of Agricultural Sciences. The cotton (TM-1) seeds were germinated on a wet filter paper for 3 days at 28 °C, and then transferred to a liquid culture medium [42]. At the 3-leaf stage, the seedlings were treated with 10% PEG 6000 and 300 NaCl; the true leaves were sampled at 0, 1, 3, 6, and 12 h of the treatment and were immediately frozen in liquid nitrogen and stored at -80 °C. The total RNA was extracted from the seedlings using RNAprep Pure Plant Kit (TIANGEN, Beijing, China), as per the manufacturer’s instructions. The first strand cDNA was synthesized using a PrimeScript® RT reagent kit (Takara, Dalian, China). SYBR Premix Ex TaqTM II (Takara) was used for PCR amplifications. The cotton histone 3 (GenBank accession no. AF024716) was used as an internal control.

## Results

### Gene identification and homeobox domain retrieval

We used WOXs from *Arabidopsis thaliana* as queries for searching the rice, sorghum, poplar, maize, cacao, *G. arboreum*, *G. raimondii*, and *G. hirsutum* databases using blastp program and hits with e-values of 1e-5 were considered significant. In preliminary analysis, we recognized 14 candidates in rice, 14 in sorghum, 18 in poplar, 20 in maize, 14 in cacao, 26 in *G. arboreum*, 31 in *G. raimondii*, 50 in *G. hirsutum* (NAU), and 33 in *G. hirsutum* (BJI). Thereafter, PROSITE (http://prosite.expasy.org/) and InterProscan 56.0 (http://www.ebi.ac.uk/interpro/) were used to search for the HB domain in the obtained sequences and 14, 12, 12, 19, 11, 21, 20, 38, and 33 genes were confirmed as WOX family members in rice, sorghum, poplar, maize, cacao, *G. arboreum*, *G. raimondii*, *G. hirsutum* (NAU), and *G. hirsutum* (BJI), respectively (Additional file 1:Table S1). According to newly sequenced A genome database (Unpublished) by PacBio RS II [43], we found the previous annotations of Cotton_A_11936 and Cotton_A_11937 were not accurate, and the total WOX genes in *G. arboreum* should be 20 , so we used *GaWOX1* to represent them for further study. Comparing the genes from the two *G. hirsutum* genomes (NAU and BJI), we found that they were highly similar, and the genes from NAU contained all the genes from BJI; therefore, we took genes from NAU for most of the analyses (Additional file 2: Table S2). The total number of WOX genes identified in the two diploid species, *G. arboreum* (AA) and *G. raimondii* (DD), was higher than that in the tetraploid *G. hirsutum* (AADD), which is derived from hybridization of progenitors resembling *G. arboreum* and *G. raimondii*. The number of WOXs in the two diploid cotton species (*G. arboreum* and *G. raimondii*) was found to be much higher than that in cacao [11], poplar [12], *Arabidopsis* [16], sorghum [12], and rice [14], indicating that the WOX family in cotton has undergone enlargement during the evolution.

### Conserved amino residues within homeobox domains

**Fig. 1:**
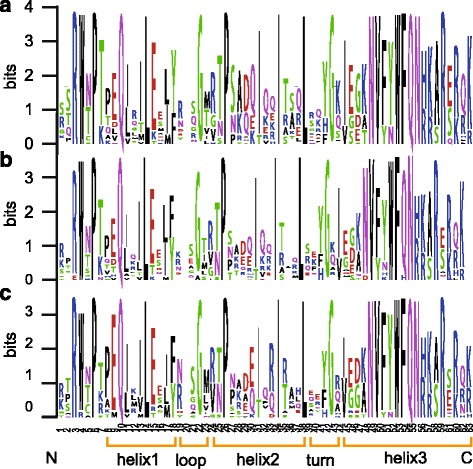
Sequence logos showing the highly conserved HD-domains in rice (**a**), *Arabidopsis* (**b**), and *G. hirsutum* (**c**).

The WOX gene family is a plant-specific clade of HD-containing superfamily, typically characterized by the presence of a conserved HB domain in the full-length sequence. To investigate the presence of homologous domain sequences and the degree of conservation of each residue in the HB domains, we performed multiple sequence alignment to generate sequence logos of the HB domain in *G. hirsutum*, *Arabidopsis*, and rice. The sequence logos revealed that the residue distribution in the HB domains was highly similar in these three plants (Fig. 1a-c). Some amino acid residues in the HB domain, for instance, Q, L, and E in helix 1, P, I, and L in helix 2, and I, N, V, F, V, W, F, Q, N, R, and R in helix 3, were highly conserved. In contrast, the amino acid residues in the loop and turn were more variable; for example, the twentieth amino acid was blank due to the insertion of an extra amino acid in some atypical HB domains (Fig. 1a-c). Therefore, our results demonstrate that the HB domain sequences from WOXs are highly conserved among the typical dicot and monocots species.

### Phylogenetic analysis and nomenclature of WOX genes

**Fig. 2:**
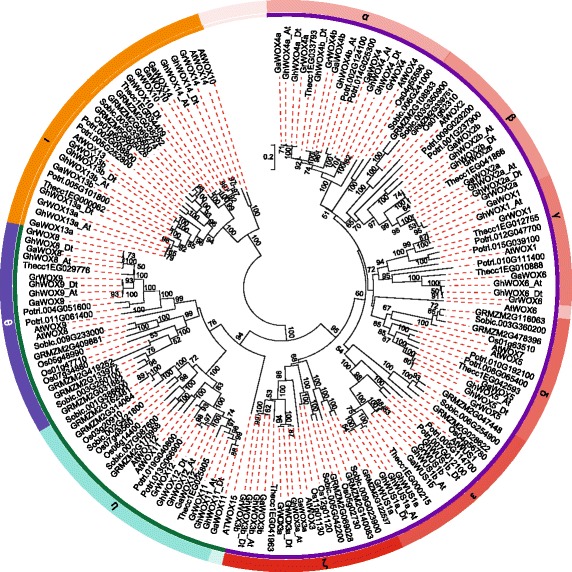
Phylogenetic tree of WOX genes indicating that WOX genes could be divided into three clades. MEGA 7.0 was used for constructing the neighbor joining (NJ) tree. The inner circle is marked in purple, green, and orange representing the WUS, intermediate, and ancient clades, respectively. Each clade was classified into sub-clades, marked in different colors on the outer circle. α to η represent the sub-clades in the WUS clade, θ and ι represent the sub-clades in the intermediate clade, and κ represents the ancient clade. The prefixes Ga, Gr, Gh, Potri, At, Os, GRMZM, Sobic, and Thecc stand for *G. arboreum*, *G. raimondii*, *G. hirsutum*, *Populus trichocarpa*, *Arabidopsis thaliana*, *Oryza sativa*, *Zea mays*, *Sorghum bicolor*, and *Theobroma cacao*, respectively. The appendices At and Dt in the upland cotton indicate the A- and D-subgenome, respectively. The bootstrap values are shown near the nodes, and only those values greater than 50 are displayed.

To determine the evolutionary relationship of WOX genes among cotton (*G. hirsutum*, *G. arboreum*, and *G. raimondii*) and other species, we constructed a phylogenetic tree by MEGA 7.0 using the NJ method. The cotton WOX genes were named based on the phylogenetic analysis. Ga, Gr, Gh, and At were used as prefixes before the names of WOX genes from *G. arboreum*, *G. raimondii*, *G. hirsutum*, and *Arabidopsis*, respectively. Moreover, following rules were also considered for the nomenclature: 1) cotton WOXs were named after their orthologs in *Arabidopsis*; 2) if there were no orthologous counterparts in *Arabidopsis*, the cotton WOXs were named based on their homologs in the same clades; and 3) “a” and “b” were appended to the gene names to distinguish the relatively recent paralogs in a particular lineage. The phylogenetic tree showed that WOX genes could be naturally classified into three clades, namely, the ancient clade, WUS clade, and intermediate clade (Fig. 2). To validate the phylogenetic tree constructed using the NJ method, we also used the minimum-evolution method to construct a tree. As show in Additional file 3: Figure. S1, WOXs were divided into three clades as shown in Fig. 2. Although, there were differences between the topologies of the two trees, the member within the subclades and the topology within the subclades were relative stable, which indicated that the NJ tree could be used for further analysis.

Compared to those of WUS and intermediate clades, the WOX genes of ancient clade were probably the earliest to diverge because the ancient clade separated the WUS and intermediate clades in the midpoint root. We found that the number of genes in the WUS clade (95) was greater than the sum of genes in ancient (28) and intermediate clades (45). To further investigate the evolutionary relationship and to predict the gene functions, we divided WOXs into nine sub-clades, named α through ι; six sub-clades were included in the WUS clade, two in the intermediate clade, and one in the ancient clade (Fig. 2; Additional file 3: Figure S1). Furthermore, we found that all the sub-clades comprised of dicot and monocot species. It is noteworthy that the genes within the sub-clades clustered with a dicot- or monocot-specific pattern. The number of WOXs in each species was variable within the sub-clades. For example, in the sub-clade ε, only one member was present in *Arabidopsis*, sorghum, rice, and cacao, each, but each of the other four species contained two members.

Compared to other species, WOX genes in cotton showed a closer relationship with that in cacao because they always clustered closely to each other in the phylogenetic tree. However, their gene number were not similar within the sub-clades, and in most cases one cacao gene corresponded to two homologous genes of *G. arboreum* and *G. raimondii*, whereas in some sub-clades, one cacao gene only had one corresponding gene in *G. arboreum* and *G. raimondii*. For example, in sub-clades θ, *Thecc1EG029776* had two orthologs in *G. arboreum* and *G. raimondii* each, whereas in sub-clade γ, *Thecc1EG012755* and *Thecc1EG010888* had only one ortholog in *G. arboreum* and *G. raimondii*, respectively.

As shown in Fig. 2 and Additional file 3: Figure S1, almost all the orthologs from A genome and At sub-genome tended to form an orthologous pair at the branch end; same was the case with the orthologs from D genome and D_t_ sub-genome, indicating that the orthologs from At-A or Dt-D had a more closer relationship.

### Gene enlargement and synteny analysis

**Fig. 3:**
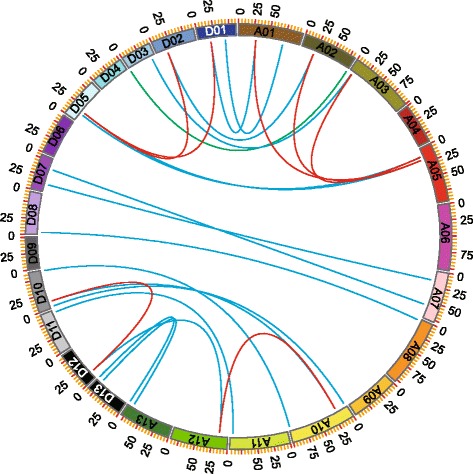
Collinearity analyses within *G. hirsutum*. The ends of blue lines link two genes from the homologous chromosome pairs in At and Dt sub-genomes, respectively, showing the orthologous pairs that diverged from a same ancestor. The green line links two duplicated genes locating in non-homologous chromosome pair in At and Dt sub-genomes. The ends of the red lines link the homologous pairs formed by segmental duplication within the At- and Dt-subgenomes. A01 to A13 represent the chromosomes in At sub-genome and D01 to D13 represent the chromosomes in Dt sub-genome.

*G. hirsutum*, the typical allotetraploid, is an ideal material for studying the effect of naturally occurring polyploidy [26]. To study the locus relationship of orthologs between the At and Dt genomes, we investigated the gene locus on chromosome and performed synteny analysis. The synteny analysis revealed that most of the WOX loci were highly conserved between the At and Dt sub-genomes (Fig. 3). We also found that 38 WOX genes in *G. hirsutum* were located on 20 chromosomes, and their distribution was uneven; for example, there were no WOX genes on chromosomes A04, D04, A06, D06, A09, and D09. Except for A02-D02, A03-D03, A11-D11, and A12-D12, the number of genes located on the chromosome in At sub-genome was the same as that on its homologous chromosome in Dt sub-genome, indicating that some genes might have been lost during the evolution or incomplete sequencing of genome might have resulted in the identification of less number of genes than were actually present; for example, *GhWOX1_At* was located on A12, but no corresponding ortholog was found in the Dt sub-genome. We further checked the genome sequence released by BJI [24]; unfortunately, we identified only one un-oriented orthologous gene (*CotAD_21819*), which was located on scaffold506.1 (Additional file 2: Table S2). Therefore, we took eleven genes in the collinearity block around *GhWOX1_At* (from *Gh_A12G2428* to *Gh_A12G2439*) to align with the cotton genome and coding sequencing database to determine its collinearity block in the Dt sub-genome. As shown in Additional file 4: Figure S2a, we found that there was a corresponding collinearity block on D12 containing 11 genes, among which *Gh_D12G2554* corresponded to *GhWOX1_At*. Thereafter, the sequences from the promoter and genomic regions (from 58,423,052 to 58,426,551 bp) were extracted from the genome to align with the A12 chromosome. As shown in Additional file 4: Figure S2b, we found that there was a deletion (998 bps) between the promoter and genomic regions on D12, which resulted in *Gh_D12G2554* losing the typical HB domain of the WOX genes (Additional file 4: Figure S2c). We also found that *GhWOX13b_At* was located on A02, but its corresponding ortholog, *GhWOX13b_Dt*, was located on D03. To confirm this, we checked *GhWOX13b* in another genome (BJI) and found two corresponding genes (*CotAD_49496* and *CotAD_38760*); however, both of them were located on the unanchored scaffold (Additional file 2: Table S2). Therefore, we used the protein sequence of *GhWOX13b* to align with the newly sequenced A genome protein database (Unpublished) by PacBio RS II [43], and found that *WOX13b* was also located on Chr02, which is the homologous chromosome of A02 (Additional file 5). We found that the collinearity around the *GhWOX1 3b_Dt* locus was conserved between the Dt03 and D03 genomes of *G. raimondii*; therefore, we presumed that a chromosomal translocation has been accomplished between Chr02 and Chr03 before cotton polyploidization forming an allotetraploid (Fig. 3; Additional file 6: Figure S3). MCSCAN was used to identify the duplicate gene types. Almost half of the WOX genes were determined to be singletons, whereas a total of 12 genes were observed to have undergone segmental duplication. No tandem and proximal duplications (in the nearby chromosomal region but not in the adjacent region) were found among WOXs. Eight WOX genes were present as dispersed genes (duplications other than segmental, tandem, and proximal) (Additional file 7: Table S3). *GhWOX4b_At* and *GhWOX4b_Dt* seem to have been more active during evolution because both of these formed two collinearity gene pairs within At and Dt sub-genome, respectively.

During the long evolutionary history, duplicated genes might have experienced functional divergence, including nonfunctionalization (loss of original functions), neofunctionalization (acquisition of novel functions), or subfunctionalization (partition of original functions) [44, 45]. To investigate whether Darwinian positive selection was associated with the WOX gene divergence after duplication, the non-synonymous divergence levels (Ka) versus synonymous divergence levels were calculated for 25 homologous pairs. Based on the Ka/Ks ratio, we can presume the selection pressure for the duplicated genes. It is generally accepted that a value of Ka/Ks = 1 indicates that the genes are pseudogenes with neutral selection, Ka/Ks < 1 indicates that the duplicated genes have a tendency to purify, and Ka/Ks > 1 shows an accelerated evolution with positive selection. In our study, we found that the Ka/Ks ratios from 15 gene pairs were smaller than 0.5 and those from seven gene pairs were between 0.5 and 1.0. Only three gene pairs (*GhWOX13a_At-GhWOX13a*, *GhWOX13b_At-GhWOX13b_Dt*, and *GhWUS1b_At-GhWUS1b_Dt*) had Ka/Ks larger than 1, and these gene pairs might have experienced relatively rapid evolution following duplication (Table 1). Because most of the Ka/Ks values were smaller than 1.0, we presumed that the Cotton WOX gene family has undergone strong purifying selection pressure with limited functional divergence that occurred after segmental duplications and whole genome duplication (WGD).

**Table 1:** The Ka and Ks values for homologous pairs

Paralogous pairs	Ka	Ks	Ka/Ks
GhWOX11_At-GhWOX11_Dt	0.007	0.036	0.192
GhWOX12_At-GhWOX12_Dt	0.004	0.031	0.119
GhWOX13a_At-GhWOX13a_Dt	0.008	0.006	1.456
GhWOX13b_At-GhWOX13b_Dt	0.008	0.006	1.323
GhWOX14_At-GhWOX14_Dt	0.031	0.044	0.716
GhWOX2a_At-GhWOX2a_Dt	0.019	0.036	0.515
GhWOX2b_At-GhWOX2b_Dt	0.007	0.072	0.101
GhWOX3a_At-GhWOX3a_Dt	0.023	0.026	0.900
GhWOX3a_At-GhWOX3b_At	0.189	0.490	0.386
GhWOX3b_At-GhWOX3b_At	0.011	0.031	0.346
GhWOX4_At-GhWOX4_Dt	0.010	0.034	0.285
GhWOX4a_At-GhWOX4a_Dt	0.011	0.026	0.424
GhWOX4b_At-GhWOX4_At	0.144	0.361	0.400
GhWOX4b_At-GhWOX4a_At	0.124	0.463	0.267
GhWOX4b_At-GhWOX4b_Dt	0.006	0.012	0.520
GhWOX4b_Dt-GhWOX4_Dt	0.133	0.366	0.363
GhWOX4b_Dt-GhWOX4a_Dt	0.097	0.456	0.213
GhWOX5_At-GhWOX5_Dt	0.002	0.042	0.052
GhWOX6_At-GhWOX6_Dt	0.020	0.025	0.803
GhWOX8_At-GhWOX8_Dt	0.012	0.049	0.243
GhWOX9_At-GhWOX9_Dt	0.009	0.014	0.619
GhWUS1a_At-GhWUS1b_At	0.144	0.438	0.329
GhWUS1a_At-GhWUS1a_Dt	0.017	0.020	0.834
GhWUS1a_Dt-GhWUS1b_Dt	0.141	0.454	0.310
GhWUS1b_At-GhWUS1b_Dt	0.003	0.000	99.042

The TEs are spread throughout the genome, and many of them are located in the vicinity of host genes [46]. Under abiotic or biotic stress, the TEs can be activated or repressed [47]. To explore whether TEs were involved in the WOX family expansion, we used *de novo* prediction and homolog search methods to identify the TEs in the whole genome, and the TEs close to the WOX genes were taken out. As show in Table 2, when checking the 2,000-bp region around the gene locus of WOX, only two retroelements (L1 and Caulimovirus) were found (Additional file 8: Table S4). We then broadened the scanning region to 10,000 bp upstream and downstream of the genes, respectively, and thirty one TEs were identified including five DNA transposons and 26 retroelements. All the five DNA transposons belonged to CMC-EnSpm family, whereas the retroelements were made up of Caulimovirus (1), Copia (13), and Gypsy (12) (Additional file 9: Table S5). Upon further inspection of the distribution of TEs within the 2,000-bp region, only one L1 was identified downstream of *GhWOX4_Dt* and one LTR/Copia was found to be located downstream of *GhWOX2a_At*. Within the 10,000-bp region around the gene locus, four CMC-EnSpm TEs were found to be located upstream of *GhWOX4a_Dt*, one CMC-EnSpm was located downstream of *GhWOX8_At*, three Copia elements were located downstream of *GhWOX13a_Dt* and *GhWOX2a_Dt*, two Copia TEs were located upstream of *GhWOX4_At* and *GhWOX6_At*, one Copia was located downstream of *GhWOX3a_At*, *GhWUS1b* and *GhWOX3a_Dt*, five Gypsy TEs were located upstream and downstream of *GhWOX13a_Dt*, three Gypsy TEs were located downstream of *GhWOX4_At*, two Gypsy TEs were located downstream of *GhWOX4_At*, and one Gypsy element was located downstream of *GhWOX13a_At* and *GhWOX3b_At*. We noticed that most of the TEs correlated with the presence of duplicated genes, which suggested that TEs, especially the retroelements, played important roles in the WOX family expansion. Compared to TEs, simple repeat sequences are more abundant, most of which locate downstream or upstream of the genes, and only 39 were located in the genomic region. The length of simple repeat sequence was very variable, which might play important roles in the divergence of gene function after duplication.

**Table 2:** The TEs around the WOX gene locus

Type	Number of elments	Length occupied	Percentage of sequence (%)	Number of elments	Length occupied	Percentage of sequence (%)
	10,000 bp region			2000 bp region		
DNA transposons	5	1264 bp	0.16	0	0 bp	0.00
CMC-EnSpm	5	1264 bp	0.16	0	0 bp	0.00
MULE-MuDR	0	0 bp	0.00	0	0 bp	0.00
PIF-Harbinger	0	0 bp	0.00	0	0 bp	0.00
TcMar-Pogo	0	0 bp	0.00	0	0 bp	0.00
hAT	0	0 bp	0.00	0	0 bp	0.00
hAT-Ac	0	0 bp	0.00	0	0 bp	0.00
hAT-Charlie	0	0 bp	0.00	0	0 bp	0.00
hAT-Tag1	0	0 bp	0.00	0	0 bp	0.00
hAT-Tip100	0	0 bp	0.00	0	0 bp	0.00
Retroelements	31	15,657 bp	1.92	2	1400 bp	0.69
LINE:	5	1501 bp	0.18	1	373 bp	0.18
L1	5	1501 bp	0.18	1	373 bp	0.18
LTR:	26	14,156 bp	1.74	1	1027 bp	0.51
Caulimovirus	1	126 bp	0.02	0	0 bp	0
Copia	13	8097 bp	0.99	1	1027 bp	0.51
Gypsy	12	5933 bp	0.73	0	0 bp	0.00
RC:	0	0 bp	0.00	0	0 bp	0.00
Helitron	0	0 bp	0.00	0	0 bp	0.00
Low_complexity	6	257	0.03	0	0 bp	0.00
Simple_repeat	559	23,669	2.91	173	6793	3.35
Unspecified	68	24,602	3.02	7	1825	0.90

### Analysis of gene structure and homeodomain location

**Fig. 4:**
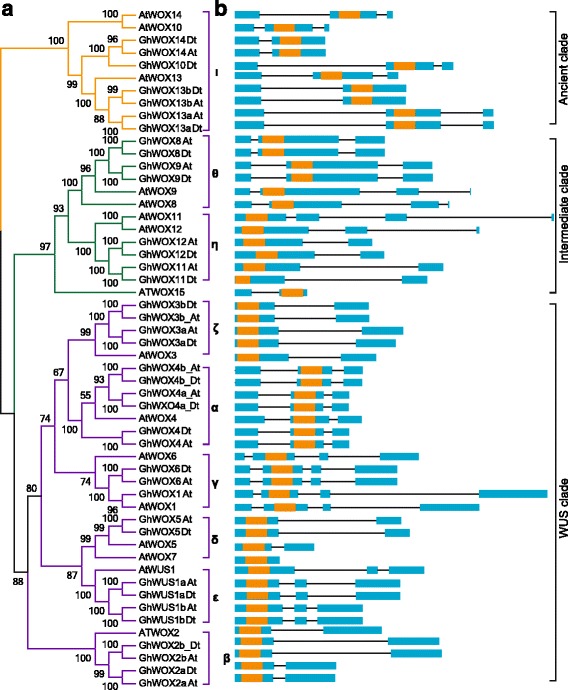
Comparison of the gene structures between *A. thaliana* and *G. hirsutum*. **a** NJ tree analysis of *A. thaliana* and *G. hirsutum*. Orange, green, and purple in the phylogenetic tree represent the ancient, intermediate, and WUS clades. **b** The number, length, and position of exons and introns within WOX genes. Boxes indicate the exons and black lines indicate the introns. The orange boxes represent the HB domains.

Gene structure is closely related to its function and, together with phylogenetic analysis, it can reflect the phylogenetic relation among the WOX genes. To further study the phylogenetic relationship between *Arabidopsis* and *G. hirsutum*, an NJ tree was generated with MEGA 7.0 using *Arabidopsis* and cotton WOX protein sequences (Fig. 4a), and positions of exons and introns in their genes were determined (Fig. 4b). Because the number of genes used for generating the phylogenetic tree described earlier (Fig. 2) was different from the one shown in Fig. 4a, the topologies of the two trees were different; however, the gene members within the sub-clades were mostly the same. As shown in Fig. 4a, WOX genes from cotton were grouped into nine sub-clades; *AtWOX15* could not be divided into any of the sub-clades. As evident from Fig. 4a, WOXs in α, ε, and ζ sub-clades of cotton might have undergone duplication because one *Arabidopsis* WOX in these sub-clades matched more than two orthologs from cotton (Fig. 4a) and this speculation was confirmed through collinearity analysis within At and Dt sub-genomes (Fig. 3). The exon number in *Arabidopsis* ranged from one to four, with the average being 2.9375, and in cotton, it ranged from two to four, with an average of 2.7297. In general, the gene structures among most of the sub-clades were conserved; for instance, in ζ, δ, and β sub-clades, the members in each sub-clade consisted of two exons. On comparing the exons in the orthologs from the same position in *Arabidopsis* and cotton, we found that the intron lengths were more divergent between the two; for example, the intron length of *AtWOX5* was much shorter than that of its ortholog in cotton. In the θ sub-clade, 6-bp-long exons were found at the fourth exon locus of *AtWOX8* and *AtWOX9* but they were not found in *GhWOX8*_*At*/*Dt* and *GhWOX9*_*At*/*Dt*, which might be due to an intron insertion in the third exon of the ancestor of *AtWOX8* and *AtWOX9* or these exons might have been lost in cotton during the evolution. We noticed that the intron lengths of *GhWOX5_At* and *GhWOX5_Dt* were different. Therefore, *GhWOX5* genomic sequences were extracted from the chromosome sequences to perform multiple alignments. We found many indels in the genomic sequence and statistically analyzed the indels more than 2 bps in length. The result showed that the introns of *GhWOX5* in the At and Dt sub-genomes were less conserved than the exons. This was because of the fact that of the eight indels present in the genomic sequences, seven were located in the introns and only one was present in the exons (Additional file 10: Figure S4). The typical conserved domains (HB domain) are marked in the exons with orange color. The lengths of HB domain ranged from 177 to 183 bp, and were considerably conserved. The HB domain locus in the same sub-clade was significantly conserved; for example, in α, γ, θ, and ι sub-clades, the HB domains were located in the second exon, and in δ, ε, ζ, β, and η sub-clades, they were located in the first exon (Fig. 4b). Only *GhWOX10_Dt* was used in our study, and its ortholog in At (*Gh_A11G2876*) was observed to have lost the third exon and part of the second exon, which resulted in the HB domains being incomplete. Further investigation revealed a point mutation at 1433 bp, turning ‘CAG’ to ‘TAG’, which lead to a premature termination of *Gh_A11G2876* protein (Additional file 11: Figure S5). To validate the above results, we searched the BJI genome; however, no corresponding orthologs were identified. Therefore, we used the RNA-seq aligned data (Bam file) to verify the result. As shown in Additional file 12: Figure S6, we found the clean reads mapped to the reference genome very well, and according to the mapping rate, we clearly found that there were two introns in *GhWOX10_Dt*. However, there was only one intron in *Gh_A11G2876*. We enlarged the mapping results near the mutation site and clearly saw that *Gh_A11G2876* acquired a termination codon at 92,69,533 bp. The point mutant might have affected the *Gh_A11G2876* gene structure, indicating that *Gh_A11G2876* might have a function different from that of *GhWOX10_Dt* or might have lost its biological function.

### Gene expression pattern in different tissues and under multiple stresses

**Fig. 5:**
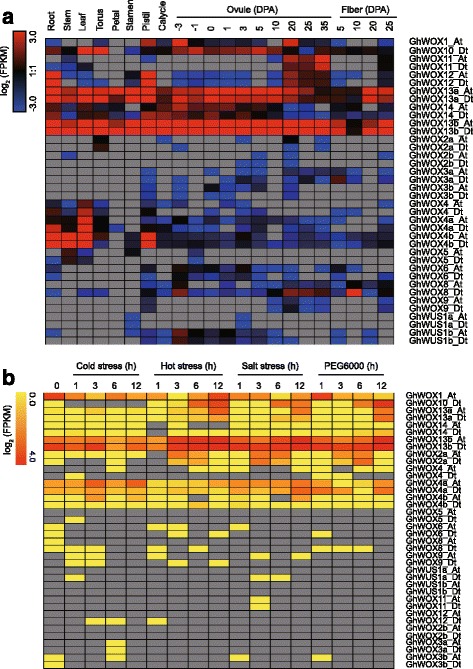
Pattern of gene expression in different tissues (**a**) and under different stresses (**b**). In (**b**) true leaves were harvested at 0, 1, 3, 6, and 12 h after the treatment.

Because gene expression is associated with the biological function, we inspected the expression patterns of different WOX genes. RNA-seq data were downloaded from NCBI and analyzed. As shown in Fig. 5a, we found that WOXs were widely expressed in the vegetative (root, stem, and leaf) and reproductive (torus, petal, stamen, pistil, calycle, and -3, -1, 0, 1, 3, 5, 10, 20, 25 and 35 days post-anthesis (DPA) ovule) tissues as well as in the fiber (5, 10, 20, and 25 DPA), indicating that WOXs have diverse biological functions and work in different tissues. We found that some WOXs did not express in the vegetative tissues and had a very low expression levels in the reproductive tissues. For instance, we could not detect *GhWOX2_At*/*Dt*, *GhWOX3a_At*/*Dt*, and *GhWOX3b_At*/*D*t expression in root, stem, and leaf, and very low expression levels were detected in some of the reproductive tissues. On comparing the expression patterns of the orthologs between At and Dt, we found that the expression patterns and the levels of expression of the two were not always the same; for example, *GhWOX8_Dt* was expressed in root and leaf but *GhWOX8_At* was not. *GhWOX8_Dt* had higher expression levels in 20, 25, and 35 DPA ovule and 10 DPA fiber compared to that in *GhWOX8_At. GhWOX13a_At*/*Dt* and *GhWOX13b_At*/*Dt* were not only close to each other in the phylogenetic tree (Fig. 2 and Fig. 4a), but also had similar expression patterns (Fig. 5), suggesting that they have a similar biological function.

Cotton faces multiple abiotic stresses during its growth and development. Therefore, a comprehensive analysis of the expression pattern of WOXs was performed in the present study. No obvious changes in the expression levels were observed for more than half of the WOXs under hot, cold, salt, and PEG 6000 conditions. *GhWOX10_Dt* responded to multiple stress treatments. The expression of *GhWOX13a_At*/*Dt* and *GhWOX13b_At*/*Dt* was strongly induced by multiple stresses, indicating that they might take part in response to stress and their expression might be regulated by stress.

### Examination of the expression of WOX genes by qPCR

**Fig. 6:**
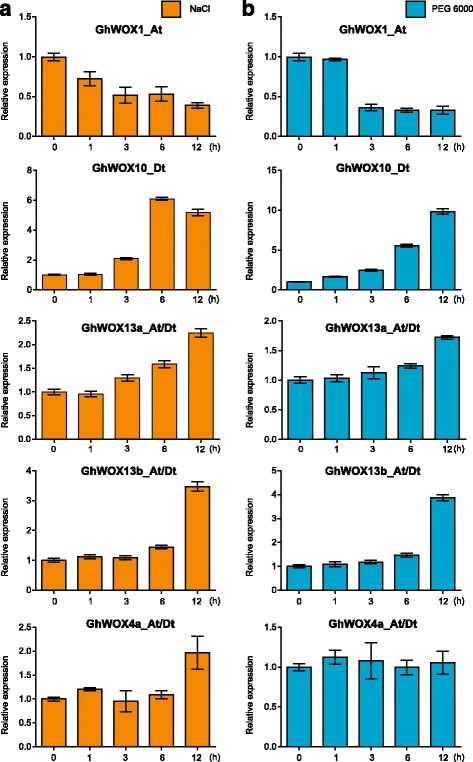
Expression levels of WOXs under NaCl (**a**) and PEG6000 (**b**) treatment . Error bars represent SD of three independent experiments

To verify the expression profiles of the WOX genes obtained by RNA-seq data, qPCR was performed using the leaves from plants treated with PEG6000 and NaCl. Eight genes, including three orthologous pairs, that were presumed to be highly expressed under PEG6000 or NaCl treatment based on the RNA-seq data, were selected for qPCR, (Fig. 5b; Fig.6). It was difficult to distinguish between the orthologous pairs by qPCR because the sequences of the orthologous genes (*GhWOX13a_At-GhWOX13a_Dt*, *GhWOX13b_At-GhWOX13b_Dt*, and *GhWOX4a_At-GhWOX4a_Dt*) were highly similar. Therefore, we designed primers to amplify the orthologous pairs together (Additional file 13: Table S6). The pattern of gene expression assessed by qPCR showed a similar tendency with that detected using the RNA-seq data, as shown in Fig. 5b and Fig. 6. The expression of *GhWOX1_At* was down-regulated under PEG 6000 and NaCl treatment. In contrast, the expression levels of *GhWOX10_Dt*, *GhWOX13b_At*/*Dt* and *GhWOX13a_At*/*Dt* were increased under both PEG 6000 and NaCl treatment, indicating that they might play vital roles in the stress response and could be the candidate genes for further study on cotton stress biology.

## Discussion

Previous analysis of the WOX gene family has been performed in rice, sorghum, maize, *Arabidopsis*, and poplar [2]. However, there were no reports on the analysis of this family in cotton. In this study, we performed a comprehensive identification of WOX genes in *G. hirsutum*, *G. arboreum*, and *G. raimondii*, mainly focusing on the allotetraploid cotton *G. hirsutum*, with the aim of understanding the roles of this gene family in cotton, in future studies.

### Cotton WOX family underwent enlargement during the evolution

Upland cotton is one of the most important cash crops worldwide, providing more than 90% of the natural renew fiber for textile industry. It is also an ideal material for studying the effects of natural polyploidy [48]. A-genome and D-genome diploid cotton were native to Africa and Mexico, respectively, and they diverged about 5–10 million years ago. About 1–2 million years ago, A-genome cotton resembling *G. arboreum* and *G. herbaceum* hybridized with D-genome cotton resembling *G. raimondii*, which was followed by chromosome doubling, and the eventual formation of nascent AtDt allopolyploid, including the upland cotton [22, 48].

A total of 38 WOX genes were identified in the upland cotton, which is more than the number of this gene reported in most of the other species whose genome has been sequenced [4]. One of the most important features was that the upland cotton experienced polyploidization, resulting in WGD. The At and Dt donors of the upland cotton are close relatives, with almost equal number of orthologs; therefore, the nascent duplication resulted in a number of WOX doublings [49]. Polyploidization is an important event during the evolution of flowering plants that might play important roles in the adaptation of plants to new environment [50]. Although the total number of upland cotton dramatically increased, gene loss also happened during the evolution of upland cotton, as evident from the comparison of the number of genes in At or Dt genomes with that in A- (*G. arboreum*) or D-genome (*G. raimondii*). Gene loss always happened during the rapid arrangement of genomic sequences after hybridization and following chromosome doubling during polyploidization [51]. Compared to the paleopolyploid maize and *Brassica*, cotton displays fewer changes in their genomic sequences [52, 53].

Although polyploidy was the main contributor in duplication, segmental duplication has also been responsible for the increase in the WOX gene number. Segmental duplication is one of the most important impetuses for evolution; it occurs most frequently in plants since most plants are diploidized polyploids and retain large amount of duplicated chromosomal blocks within their genomes [54]. The WRKY genes in soybean underwent large scale segmental duplication, with the duplicated genes exhibiting differential expression and functional divergence [55]. Heat shock proteins play important roles in response to drought stress in sesame; evolutionary analysis showed that segmental duplication was the primary force promoting the expansion of heat shock promoting genes [56]. In our study, 12 out of 38 genes were associated with segmental duplication, indicating that segmental duplication played significant roles in the expansion of WOX genes, and differential expression suggested that the duplicated genes might have experienced functional divergence.

Although we did not find tandem duplication in our study, it is also a basic contributor to gene expansion, which arises from unequal crossing over, chromosomal anomalies, transposon insertions, and other reverse-transcriptase-mediated processes [57]. After duplication, the new gene would be redundant with the previously existing one. The redundancy has been considered a driving force for evolutionary innovation [57]. Some models have been proposed for understanding the gene duplication; these include the neofunctionalization, DDC-subfunctionalization, escape from adaptive conflict (EAC), and dosage-balance models, which provide a theoretical framework for further studying the process [57].

### Cotton WOXs have been highly conserved during the evolution

WOX is a plant-specific sub-clade with few members of HD-containing superfamily, which consists of a leucine zipper (HD-ZIP), plant homeodomain associated to a finger domain (PHD finger), the distinctive Bell domain (Bell), zinc finger associated to a homeodomain (ZF-HD), knotted-related homeobox (KNOX), and WUSCHEL-related homeobox (WOX) [58]. Previous studies have shown that HDs (HB-domain) might have diverged before the separation of the branches forming plants, animals, and fungi [59]. The members of HD-containing superfamily were different not only in the sequence of HB domain, but also in their size, HB location, other related domains as well as in their structures [58]. WOX predominantly has HB domain, and can be distinguished by the phylogenetic relatedness of its homeodomains [1]. It has been reported that HB domain contains some conserved amino acids located in the helices; for example, it contains Q, L, and Y in helix 1 and I, V, W, F, N, K, R, and R in helix 3 [2]. Our results demonstrate that the above amino acids were also conserved in cotton. Previous studies have indicated that the amino acids in loops were less conserved [2], which is in consonance with our results. Previous studies have also shown that WOXs could be divided into three clades [1, 2, 60], as was observed in the present study for cotton (Fig. 2). In a study by Zhang et al., WOX family was further classified into nine sub-clades, as was the case in the present study [2]. We observed that *AtWOX6* was not divided into any sub-clades (Fig. 2) because it had a bootstrap value lower than 50. We found that in the study by Zhang et al., *AtWOX6* was clustered into the same clade as *AtWOX1* despite the bootstrap value not supporting this classification. However, as shown in Fig. 4, we found that *AtWOX6* and *AtWOX1* might have originated from the same ancestor, a fact supported by a high bootstrap value. Therefore, we presumed that the increasing number of sequences in Fig. 2 would make the total number of sequences more divergent, resulting in *AtWOX6* being not stable between Fig. 2 and Fig. 4. Because the annotation is gradually improving, *AtWOX15* (*AT5G46010.1*) was identified as a novel member that was not reported in previous studies [1, 2]. We found that it belonged to the intermediate clade, but it could not be divided into any sub-clade because as per our definition in the present study, a new sub-clade should have at least two members. The monocot- and dicot-specific cluster pattern suggested that the main functions of WOX genes had been determined before the monocots and dicots split. Previous studies have shown that in ζ (NS/WOX3) sub-clade, the genes from monocots formed two separate branches [2], which was consistent with our results. However, we found that there were two pairs of duplicated genes (*GhWOX3a_At*-*GhWOX3b_At* and *GhWOX3a_Dt*-*GhWOX3b_Dt*) in cotton, with no orthologs in poplar, which indicated that poplar might have lost these genes during evolution or this might have resulted from an incomplete sequencing result. In the report of Zhang et al., it was thought that the subgroup B (corresponding to sub-clade α in our study) might be an ancient subgroup, because only one member from each species was present in this clade [2]. However, our data did not support this opinion because we found that *GhWOX4_At*/*Dt*, *GhWOX4a_At*/*Dt*, and *GhWOX4b_At*/*Dt* were duplicated genes (Fig. 3). Cacao and cotton diverged from a common ancestor about 18–58 million years ago, and both of them had undergone the ancient duplication event together; cotton had subsequently experienced a nascent duplication event again [25], our data also indicated that cacao and cotton were close relative and probably derived from the same ancestor. One cacao WOX gene should theoretically correspond to two orthologs in the diploid cotton, but in fact some genes in *G. arboreum* and *G. raimondii* might have been lost during the evolution [51]. It is generally believed that *G. hirsutum* (2n = 4x = 52, (AD)_t_) was reunited by hybridization of an A-genome species resembling *G. arboreum* (2n = 26, A_2_) with a D-genome material resembling *G. raimondii* (2n = 26, D_5_), followed by chromosome doubling [24]. Our data suggested that WOXs in A genome and At sub-genome had a common ancestor, and those in D genome and Dt sub-genome had a common ancestor, which was consistent with the above hypothesis, indicating that cotton WOXs were highly conserved during the evolution.

### Expression of duplicated WOX genes

Previous studies on the expression and function of WOX genes have indicated that the WOX family members play crucial roles in key developmental processes of plants, such as in embryonic patterning, stem-cell maintenance, and organ formation [1]. Our data (Fig. 5a) suggest that most of the WOX genes had very low expression levels or they did not express in selected tissues, the reason for which might be that WOX family members mainly function in the process of embryogenesis because of which their expression is restricted. Despite the expression levels and the number of expressed genes being different among the different tissues (Fig. 5a), except for *GhWOX8_At*/*Dt*, the orthologous gene pairs did not show A- or D-ortholog bias, which suggests that they may have conserved functions. Segmental duplication was one of the most important impetuses for increasing the diversity at the molecular level. After duplication, the coding regions could have acquired new regulatory context through the acquisition/deletion of tissue-specific enhancers and repressors, causing the spatial and/or temporal change in the expression pattern of the duplicated gene, promoting diversification of gene functions, like subfunctionalization (acquisition of part of the function of a pre-existing gene) or neofunctionalization (acquisition of a new function) [61]. *AtWOX4* works in procambium development, which is associated with vascular patterning and leaf complexity [62]. In upland cotton, we observed the expansion of *WOX4* through WGD and segmental duplication, and the duplicated genes showed different expression levels in root and stem, which indicated that these genes might have acquired subfunctionalization or a novel function [57].

In *Arabidopsis thaliana*, WOX8 protein not only positively regulates early embryonic growth, but also interacts with *AtCLE8* to promote seed growth and the overall seed size [63]. In the present study, *GhWOX8_Dt*, the ortholog of *AtWOX8* in upland cotton, was observed to express abundantly during the seed development, indicating that *GhWOX8_Dt* might have similar function in seed development as *AtWOX8* has in *Arabidopsis*. However, *GhWOX8_At* might have a divergent function in cotton seed development, because it had a different expression pattern in ovule compared to that of *GhWOX8_Dt*, suggesting that *GhWOX8s* might have experienced functional divergence after duplication and *GhWOX8_Dt* could be a key candidate gene in cotton seed development that should be studied further.

*AtWOX11* interacts with *WOX12*; these two genes are involved in the first-step of cell fate transition during the *de novo* root organogenesis [18]. *GhWOX12* was expressed specifically in root, which suggested that *GhWOX12* might play important roles in root development; however, confirmation as to whether it works together with *GhWOX11* in regulating the *de novo* root organogenesis would need future investigation. *GhWOX11* and *GhWOX12* had similar expression patterns as *GhWOX8_Dt* had in ovule; however, whether these genes have similar functions in seed development would need to be ascertained in further studies.

*AtWOX13* is a key component in the regulation of floral transition and root development, and has a high expression level during primary and lateral root initiation and development, in gynoecium, and during embryo development [64]. We found that its orthologs in cotton, *GhWOX13a_At*/*Dt* and *GhWOX13b_At*/*Dt*, were dramatically expressed in the root, in reproductive organs, as well as during the embryo development, indicating that *WOX1*3 genes in cotton and *Arabidopsis* have a conserved expression pattern, and might have a similar biological function. We also noticed that the other homologs of *WOX13* in cotton from the same sub-clade also expressed in all the assessed tissues, which suggested that the genes in the ancient clades might be more active during cotton growth and development. Although most of the reports on WOXs determined their roles in embryogenesis, they might play important roles in stress response. The mutant *hos9-1*, which was generated by a T-DNA insertion into the fourth exon of *AtWOX6*, was reported to be hypersensitive to freezing treatment [13]. Three WOXs from paper mulberry were induced by cold exposure [65]. A previous study showed that *Os08g14400* was up-regulated under desiccation and salt stress, *Os03g20910* was up-regulated under salt stress, and *Os01g63510* was down-regulated under salt stress [61]. In our study, some of the WOX genes were also induced by different stress, indicating that they may meditate the stress response. We found that the duplicated genes *GhWOX4_At*/*Dt*, *GhWOX4a_At*/*Dt*, and *GhWOX4b_At*/Dt responded differently when exposed to different stresses, suggesting that they have different functions under different stresses. *GhWOX13b_At*/*Dt* was induced by multiple stresses, indicating that *GhWOX13b_At*/*Dt* might be the node for multiple stress regulation. Although WOX genes were shown to have different expression levels under multiple stresses, there are no reports on the validation of the function of cotton WOXs in stress. Therefore, future studies should focus on determining the function of WOX genes in stress response.

## Conclusion

Previous studies have illustrated that members of the WOX gene family play significant roles in the regulation of plant growth and development by determining the cell fate. The results of the present study indicate that WOX genes are highly conserved among cotton and other plant species. Furthermore, whole genome and segmental duplication have probably been the two major ways for gene amplification during the expansion of the WOX family in upland cotton. Moreover, the duplicated genes in cotton seem to have experienced functional divergence because the duplicated genes showed different expression patterns in different tissues and organs. In addition, some WOX gene members are likely to be involved in the mediation of stress response. Our results will not only deepen the understanding of the evolutionary processes of cotton WOX genes, but would also be helpful in formulating further functional genomic studies of WOX gene family in cotton.

## Additional files

Additional file 1: Table S1. Information on the gene exons used in this study. (XLSX 46 kb)

Additional file 2: Table S2. The correspondence between WOX genes from two versions of the *G. hirsutum* genome. (DOCX 22 kb)

Additional file 3: Figure S1. Phylogenetic tree of WOX genes indicating that WOX genes could be divided into three clades. MEGA 7.0 was used for constructing the tree using the minimum-evolution method. The inner circle is marked in purple, green, and orange representing the WUS, intermediate, and ancient clades, respectively. The bootstrap values are shown near the nodes, and only those values greater than 50 are displayed. (PDF 855 kb)

Additional file 4: Figure S2. Comparative analysis of *GhWOX1_At* and *Gh_D12G2554*. (a) The collinearity analysis between chromosome A12 from 86,759,600 bp to 86,860,416 bp and D12. (b) An indel on D12 resulted in coding sequence that is divergent in *GhWOX1_At* and *Gh_D12G2554*. (c) Amino sequence alignment of *GhWOX1_At* and *Gh_D12G2554*). (PDF 599 kb)

Additional file 5: Blastn result showing the ortholog of *WOX10* in *G. arboreum* located on Chr02. (TXT 6 kb)

Additional file 6: Figure S3. Location of cotton WOX genes on chromosomes. The red dotted lines link the orthologs located on At and Dt. (PDF 508 kb)

Additional file 7: Table S3. Information on duplicated genes. (DOCX 18 kb)

Additional file 8: Table S4. Identification of repeat sequences in the region 2,000 bp upstream to 2,000 bp downstream of WOX genes. (TXT 20 kb)

Additional file 9: Table S5. Identification of repeat sequences in the region 10,000 bp upstream to 10,000 bp downstream of WOX genes. (TXT 75 kb)

Additional file 10: Figure S4. Multiple sequence alignment of *GhWOX5_At* and *GhWOX5_Dt*. (PDF 47 kb)

Additional file 11: Figure S5. Multiple sequence alignment of *Gh_A11G2876* and *Gh_WOX10_Dt*. (PDF 51 kb)

Additional file 12: Figure S6. Mapping reads around *GhWOX10_Dt* (a, b) and *Gh_A11G2876* (c, d). (PDF 3605 kb)

Additional file 13: Table S6. The primers used in present study for qPCR. (DOCX 16 kb)

## Abbreviations

BLAST: Basic Local Alignment Search Tool
CUC: Cupshaped cotyledon
DPA: Days post-anthesis
EAC: Escape from adaptive conflict
FPKM: Fragments per kilobase million
*G. arboreum*: *Gossypium arboreum*
*G. hirsutum*: *Gossypium hirsutum*
*G. raimondii*: *Gossypium raimondii*
HB: Homeobox
HD: Homeodomain
NJ: Neighbor-joining
PXY: Phloem intercalated with xylem
TE: Transposable elements
WGD: Whole genome duplication
WOX: WUSCHEL-related homeobox
