# Supplementary material for: Genome-wide analysis of WOX genes in upland cotton and their expression pattern under different stresses
Source: BMC Plant Biol. 2017 Jul 6;17:113. doi: 10.1186/s12870-017-1065-8 (PMC5501002; doi:10.1186/s12870-017-1065-8)
Supplement: Supplementary file 2 — The correspondence between WOX genes from two versions of the G. hirsutum genome. (DOCX 22 kb) [file 12870_2017_1065_MOESM2_ESM.docx]

|  | Gene name | gene locus | Chr | direction | start | end | Gene locus | Chr | direction | start | end |
| --- | --- | --- | --- | --- | --- | --- | --- | --- | --- | --- | --- |
| 1 | GhWOX1_At | CotAD_21819 | scaffold506.1 | - | 684026 | 686623 | Gh_A12G2429 | A12 | - | 86768862 | 86771458 |
| 2 | GhWOX11_At | CotAD_04402 | At_chr8 | + | 76675940 | 76677667 | Gh_A13G1402 | A13 | - | 70929455 | 70931186 |
| 3 | GhWOX11_At | CotAD_30787 | At_chr13 | - | 20593310 | 20595045 | Gh_A13G1402 | A13 | - | 70929455 | 70931186 |
| 4 | GhWOX12_At | CotAD_34495 | At_chr1 | - | 3739452 | 3740594 | Gh_A11G2676 | A11 | + | 88906993 | 88908135 |
| 5 | GhWOX13a_At | CotAD_70151 | Dt_chr1 | + | 86993580 | 86996168 | Gh_A07G1563 | A07 | + | 57203829 | 57205977 |
| 6 | GhWOX13a_Dt | CotAD_63006 | Dt_chr1 | - | 84152526 | 84154671 | Gh_D07G1730 | D07 | + | 38156360 | 38158511 |
| 7 | GhWOX13b_At | CotAD_49496 | scaffold2568.1 | + | 89153 | 90574 | Gh_A02G1705 | A02 | + | 83237195 | 83238616 |
| 8 | GhWOX13b_Dt | CotAD_38760 | scaffold1977.1 | - | 129512 | 130936 | Gh_D03G0014 | D03 | - | 130381 | 131805 |
| 9 | GhWOX14_At | CotAD_07344 | scaffold72.1 | - | 1857884 | 1858636 | Gh_A08G0247 | A08 | - | 2688010 | 2688762 |
| 10 | GhWOX14_Dt | CotAD_50682 | scaffold1865.1 | + | 234229 | 234978 | Gh_D08G0336 | D08 | - | 3380186 | 3380935 |
| 11 | GhWOX2a_At | CotAD_64898 | scaffold1775.1 | + | 132674 | 133508 | Gh_A07G0882 | A07 | - | 15552934 | 15553768 |
| 12 | GhWOX2a_Dt | CotAD_49908 | Dt_chr1 | + | 46852047 | 46852887 | Gh_D07G0951 | D07 | - | 12710159 | 12710999 |
| 13 | GhWOX2b_At | CotAD_69754 | scaffold4908.1 | + | 6391 | 8112 | Gh_A13G1983 | A13 | - | 79347577 | 79349298 |
| 14 | GhWOX2b_Dt | CotAD_04633 | At_chr4 | + | 36798930 | 36800628 | Gh_D13G2382 | D13 | - | 59943802 | 59945500 |
| 15 | GhWOX3a_At | CotAD_09560 | At_chr9 | + | 52963986 | 52965384 | Gh_A05G0852 | A05 | - | 8497547 | 8498943 |
| 16 | GhWOX3a_Dt | CotAD_01720 | Dt_chr9 | - | 58888740 | 58890077 | Gh_D05G3885 | scaffold4075_D05 | + | 13293 | 14630 |
| 17 | GhWOX3b_At | CotAD_60193 | At_chr5 | - | 5819375 | 5820489 | Gh_A03G0301 | A03 | + | 5077293 | 5078407 |
| 18 | GhWOX3b_Dt | CotAD_29562 | Dt_chr3 | - | 21734383 | 21735493 | Gh_D03G1275 | D03 | - | 40638897 | 40640007 |
| 19 | GhWOX4_At | CotAD_75974 | At_chr3 | + | 18065616 | 18066567 | Gh_A02G0851 | A02 | + | 22056095 | 22057042 |
| 20 | GhWOX4_Dt | CotAD_59256 | At_chr3 | - | 60999089 | 60999340 | Gh_D02G0901 | D02 | + | 17957326 | 17958273 |
| 21 | GhWOX4_Dt | CotAD_68050 | Dt_chr5 | + | 62747405 | 62748352 | Gh_D02G0901 | D02 | + | 17957326 | 17958273 |
| 22 | GhWOX4a_At | CotAD_69502 | At_chr2 | - | 21935268 | 21936246 | Gh_A01G0998 | A01 | + | 28211758 | 28212736 |
| 23 | GhWOX4a_At | CotAD_52695 | At_chr2 | + | 37535707 | 37536685 | Gh_A01G0998 | A01 | + | 28211758 | 28212736 |
| 24 | GhWOX4b_At | CotAD_03659 | At_chr9 | - | 42888514 | 42889582 | Gh_A05G1768 | A05 | - | 18643896 | 18644955 |
| 25 | GhWOX4b_Dt | CotAD_31335 | Dt_chr9 | - | 53462602 | 53463658 | Gh_D05G1962 | D05 | - | 18071480 | 18072536 |
| 26 | GhWOX6_At | CotAD_72593 | scaffold2593.1 | - | 64698 | 66046 | Gh_A01G1252 | A01 | - | 72702363 | 72703711 |
| 27 | GhWOX9_At | CotAD_64064 | scaffold3586.1 | - | 88370 | 90008 | Gh_A05G1334 | A05 | + | 13652171 | 13653809 |
| 28 | GhWUS1a_Dt | CotAD_27071 | Dt_chr8 | - | 32429987 | 32431363 | Gh_D12G0569 | D12 | - | 10599970 | 10601346 |
| 29 | GhWUS1a_Dt | CotAD_27072 | Dt_chr8 | - | 32427417 | 32428793 | Gh_D12G0569 | D12 | - | 10599970 | 10601346 |
| 30 | GhWUS1b_At | CotAD_21207 | At_chr11 | - | 69193777 | 69194874 | Gh_A10G0884 | A10 | + | 19135722 | 19136784 |
| 31 | GhWUS1b_Dt | CotAD_15677 | Dt_chr11 | - | 9178448 | 9179511 | Gh_D10G0866 | D10 | - | 10961089 | 10962150 |
| 32 | GhWOX4a_Dt | CotAD_37336 | Dt_chr2 | - | 37439179 | 37440156 | Gh_D01G1055 | D01 | + | 19739272 | 19740249 |
| 33 | GhWOX8_At | CotAD_00646 | scaffold26.1 | + | 737166 | 738408 | Gh_A10G0270 | A10 | - | 2379096 | 2380338 |
