# Supplementary figures and images for: Genome-wide analysis of WOX genes in upland cotton and their expression pattern under different stresses
Source: BMC Plant Biol. 2017 Jul 6;17:113. doi: 10.1186/s12870-017-1065-8 (PMC5501002; doi:10.1186/s12870-017-1065-8)

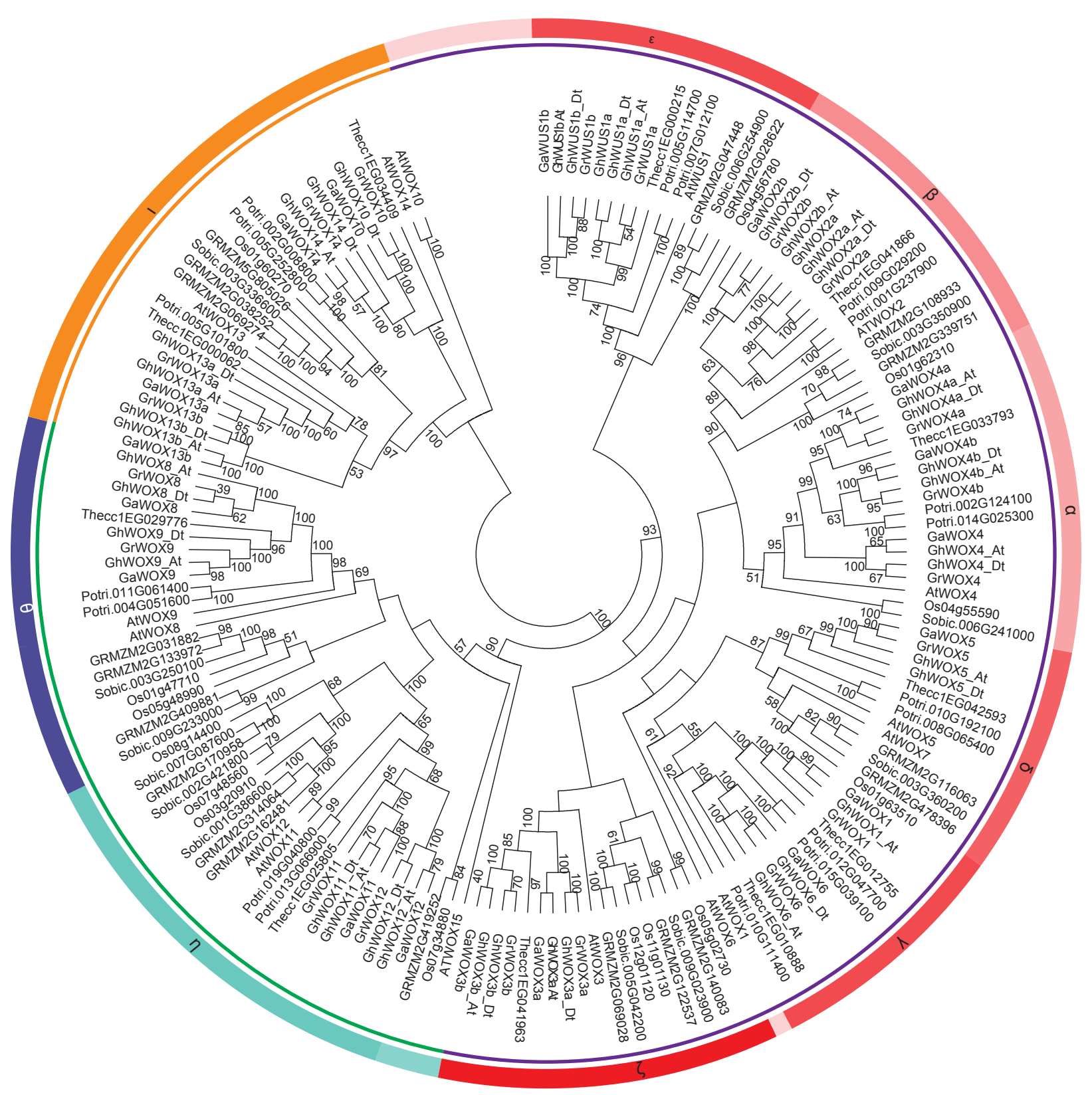

Supplement: Supplementary file 3 — Phylogenetic tree of WOX genes indicating that WOX genes could be divided into three clades. MEGA 7.0 was used for constructing the tree using the minimum-evolution method. The inner circle is marked in purple, green, and orange representing the WUS, intermediate, and ancient clades, respectively. The bootstrap values are shown near the nodes, and only those values greater than 50 are displayed. (PDF 855 kb) [file 12870_2017_1065_MOESM3_ESM.pdf]

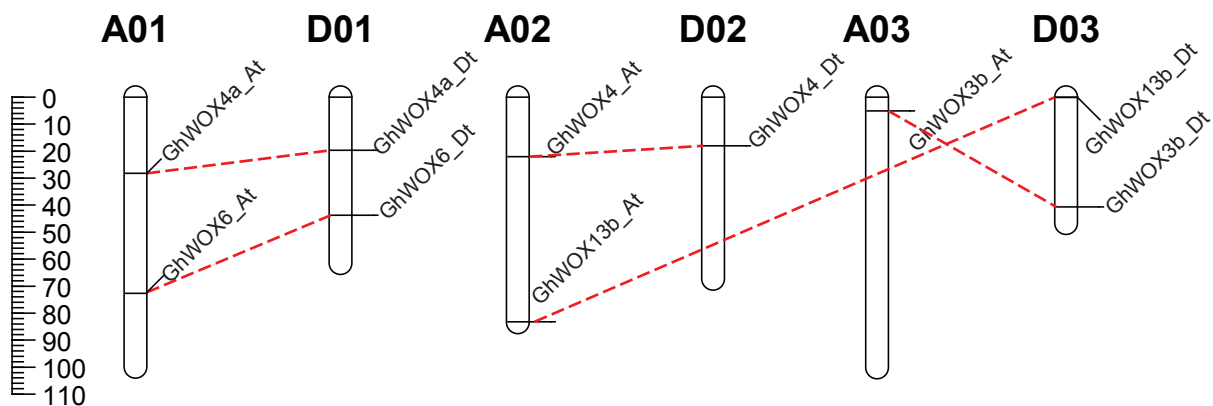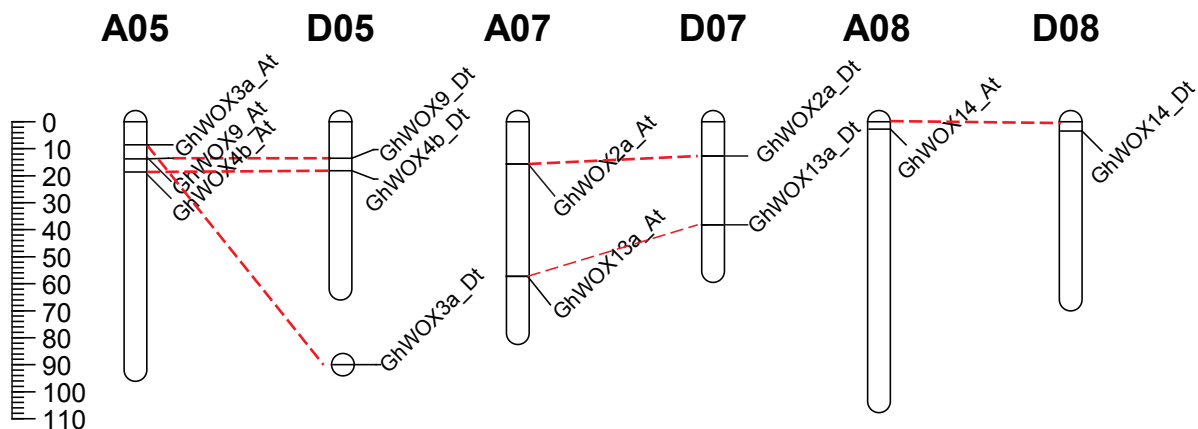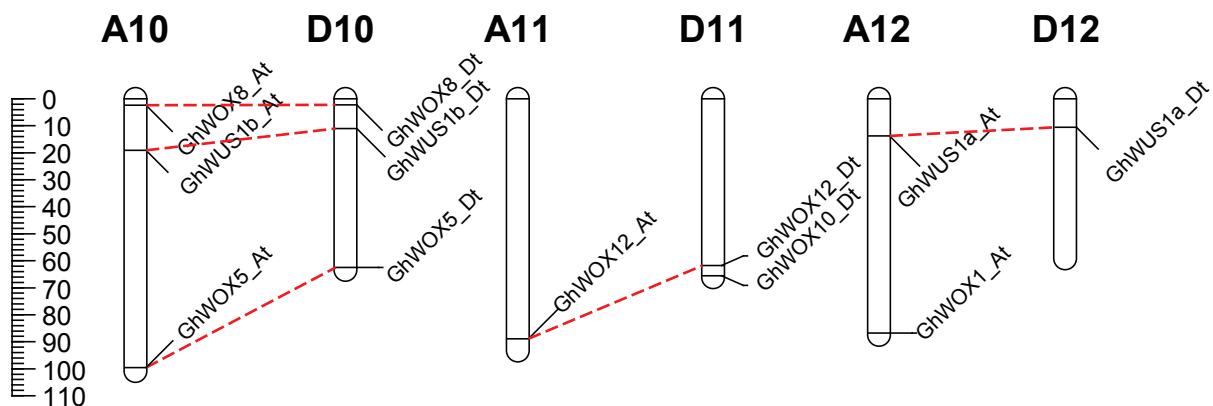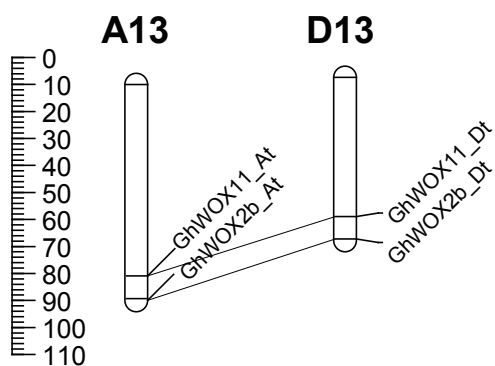

Supplement: Supplementary file 6 — Location of cotton WOX genes on chromosomes. The red dotted lines link the orthologs located on At and Dt. (PDF 508 kb) [file 12870_2017_1065_MOESM6_ESM.pdf]

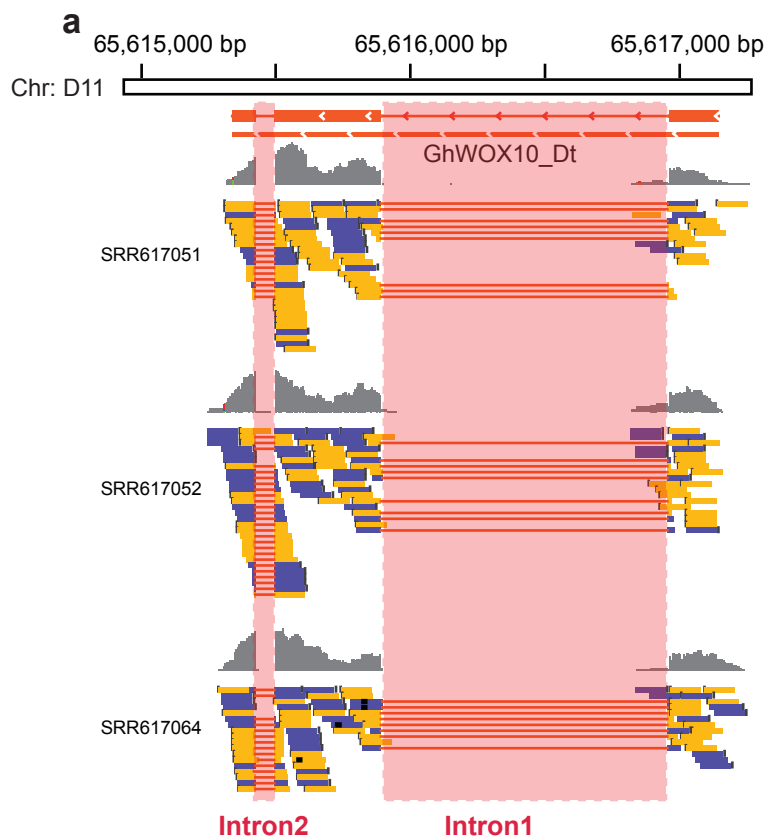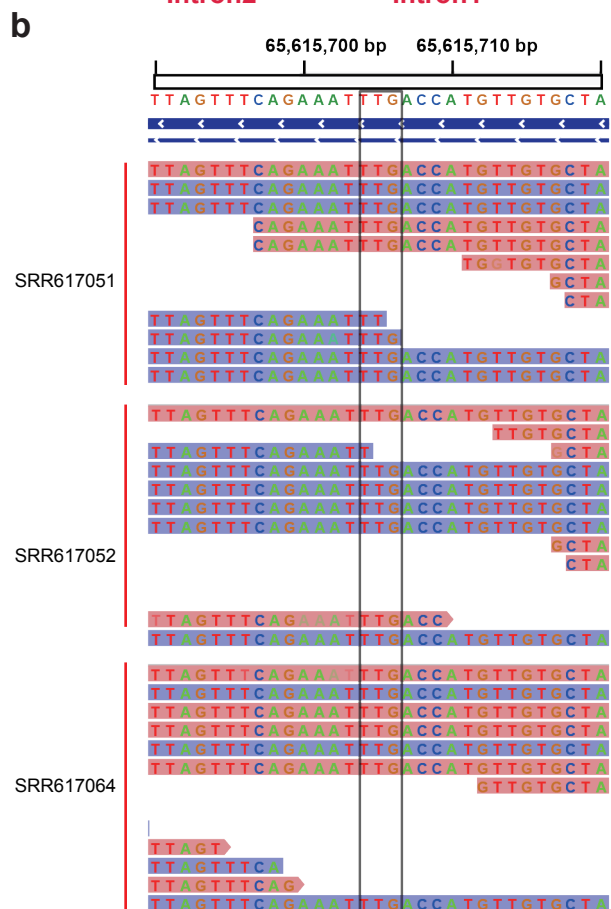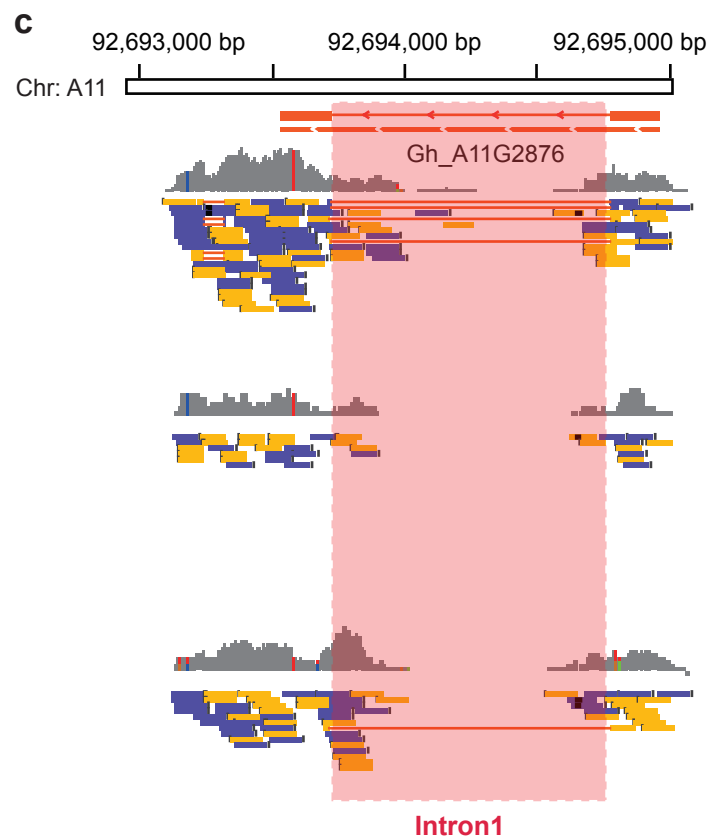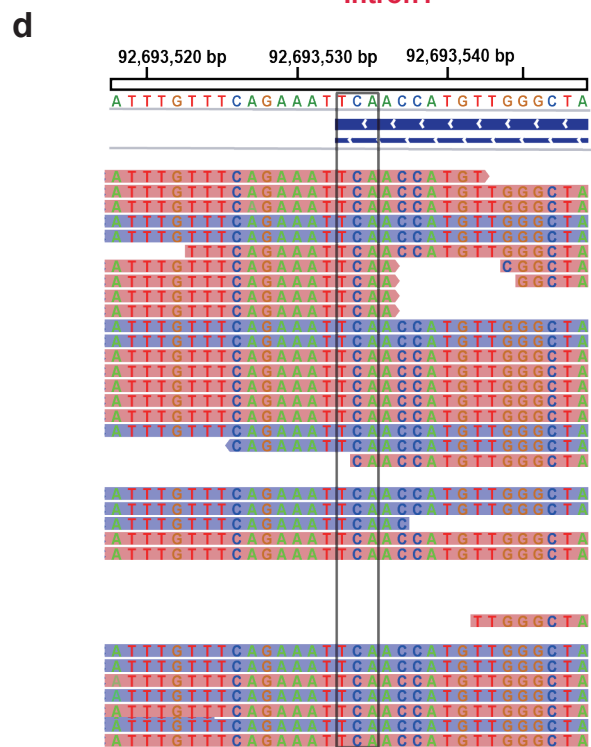

Supplement: Supplementary file 12 — Mapping reads around GhWOX10_Dt (a, b) and Gh_A11G2876 (c, d). (PDF 3605 kb) [file 12870_2017_1065_MOESM12_ESM.pdf]
