# Supplementary material for: Genome-wide analysis of WOX genes in upland cotton and their expression pattern under different stresses
Source: BMC Plant Biol. 2017 Jul 6;17:113. doi: 10.1186/s12870-017-1065-8 (PMC5501002; doi:10.1186/s12870-017-1065-8)
Supplement: Supplementary file 4 — Comparative analysis of GhWOX1_At and Gh_D12G2554. (a) The collinearity analysis between chromosome A12 from 86,759,600 bp to 86,860,416 bp and D12. (b) An indel on D12 resulted in coding sequence that is divergent in GhWOX1_At and Gh_D12G2554. (c) Amino sequence alignment of GhWOX1_At and Gh_D12G2554). (PDF 599 kb) [file 12870_2017_1065_MOESM4_ESM.pdf]

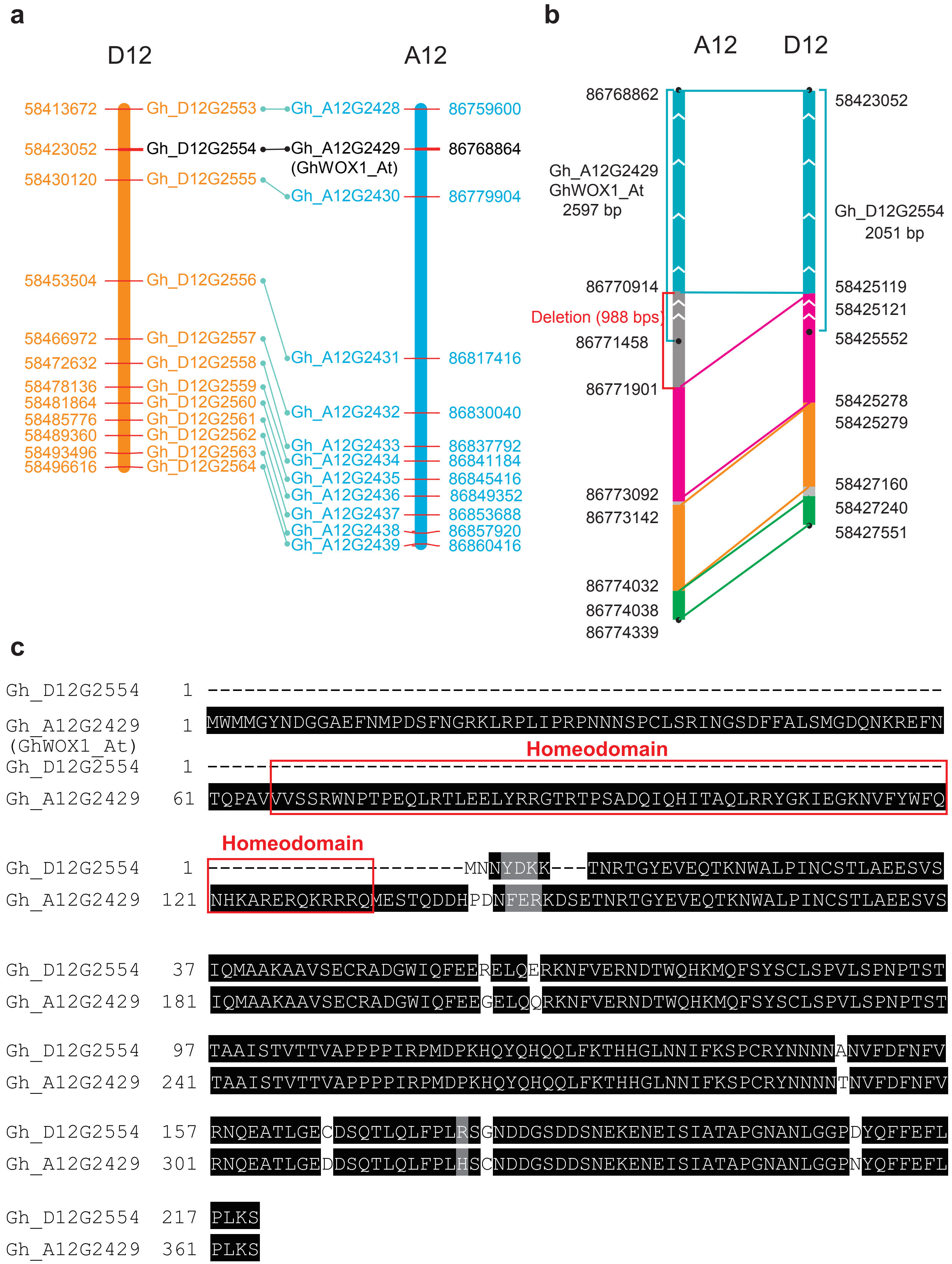
a
D12
A12
58413672
Gh_D12G2553
Gh_A12G2428
86759600
58423052
Gh_D12G2554
Gh_A12G2429
(GhWOX1_At)
86768864
58430120
Gh_D12G2555
Gh_A12G2430
86779904
58453504
Gh_D12G2556
58466972
Gh_D12G2557
Gh_A12G2431
86817416
58472632
Gh_D12G2558
Gh_A12G2432
86830040
58478136
Gh_D12G2559
58481864
Gh_D12G2560
58485776
Gh_D12G2561
58489360
Gh_D12G2562
58493496
Gh_D12G2563
58496616
Gh_D12G2564
Gh_A12G2433
86837792
Gh_A12G2434
86841184
Gh_A12G2435
86845416
Gh_A12G2436
86849352
Gh_A12G2437
86853688
Gh_A12G2438
86857920
Gh_A12G2439
86860416
b
A12
D12
86768862
58423052
Gh_A12G2429
GhWOX1_At
2597 bp
Gh_D12G2554
2051 bp
86770914
58425119
58425121
Deletion (988 bps)
86771458
58425552
86771901
58425278
58425279
86773092
58427160
86773142
58427240
58427551
86774032
86774038
86774339
c
Gh_D12G2554 1 --
Gh_A12G2429 1 MWMMGYNDGGAEFNMPDSFNGRKLRPLIPRPNNNSPCLSRINGSDFFALSMGDQNKREFN
(GhWOX1_At)
Homeodomain
Gh_D12G2554 1 --
Gh_A12G2429 61 TQPAVVVSSRWNPTPEQLRTLEELYRRGTRTPSADQIQHITAQLRRYGKIEGKNVFYWFQ
Homeodomain
Gh_D12G2554 1 ----------------------MNNYDKK---TNRTGYEVEQTKNWALPINCSTLAEESVS
Gh_A12G2429 121 NHKARERQKRRRQMESTQDDHPDNFERKDSETNRTGYEVEQTKNWALPINCSTLAEESVS
Gh_D12G2554 37 IQMAAKAAVSECRADGWIQFEERELQERKNFVERNDTWQHKMQFSYSCLSPVLSPNPTST
Gh_A12G2429 181 IQMAAKAAVSECRADGWIQFEEGELQQRKNFVERNDTWQHKMQFSYSCLSPVLSPNPTST
Gh_D12G2554 97 TAAISTVTTVAPPPPIRPMDPKHQYQHQQLFKTHHGLNNIFKSPCRYNNNNANVFDFNFV
Gh_A12G2429 241 TAAISTVTTVAPPPPIRPMDPKHQYQHQQLFKTHHGLNNIFKSPCRYNNNNTNVFDFNFV
Gh_D12G2554 157 RNQEATLGECDSQTLQLFPLRSGNDDGSDDSNEKENEISIATAPGNANLGGPDYQFFEFL
Gh_A12G2429 301 RNQEATLGEDDSQTLQLFPLHSCNDDGSDDSNEKENEISIATAPGNANLGGPNYQFFEFL
Gh_D12G2554 217 PLKS
Gh_A12G2429 361 PLKS
