# Supplementary material for: Genome-wide analysis of WOX genes in upland cotton and their expression pattern under different stresses
Source: BMC Plant Biol. 2017 Jul 6;17:113. doi: 10.1186/s12870-017-1065-8 (PMC5501002; doi:10.1186/s12870-017-1065-8)
Supplement: Supplementary file 7 — Information on duplicated genes. (DOCX 18 kb) [file 12870_2017_1065_MOESM7_ESM.docx]

**Additional file 7 Duplicated gene information**

| Gene | Gene_type | Chromosome | Gene | Gene_type | Chromosome |
| --- | --- | --- | --- | --- | --- |
| GhWOX4a_At | 4 | A01 | GhWXO4a_Dt | 4 | D01 |
| GhWOX6_At | 0 | A01 | GhWOX6_Dt | 0 | D01 |
| GhWOX4_At | 4 | A02 | GhWOX4_Dt | 4 | D02 |
| GhWOX13b_At | 1 | A02 | GhWOX13b_Dt | 1 | D03 |
| GhWOX3b_At | 4 | A03 | GhWOX3b_Dt | 0 | D03 |
| GhWOX3a_At | 4 | A05 | GhWOX3a_Dt | - | scaffold4075_D05 |
| GhWOX9_At | 0 | A05 | GhWOX9_Dt | 0 | D05 |
| GhWOX4b_At | 4 | A05 | GhWOX4b_Dt | 4 | D05 |
| GhWOX2a_At | 0 | A07 | GhWOX2a_Dt | 0 | D07 |
| GhWOX13a_At | 1 | A07 | GhWOX13a_Dt | 1 | D07 |
| GhWOX14_At | 1 | A08 | GhWOX14_Dt | 1 | D08 |
| GhWOX8_At | 0 | A10 | GhWOX8_Dt | 0 | D10 |
| GhWUS1b_At | 4 | A10 | GhWUS1b_Dt | 4 | D10 |
| GhWOX5_At | 0 | A10 | GhWOX5_Dt | 0 | D10 |
| GhWOX12_At | 0 | A11 | GhWOX12_Dt | 0 | D11 |
| GhWUS1a_At | 4 | A12 | GhWUS1a_Dt | 4 | D12 |
| GhWOX11_At | 0 | A13 | GhWOX11_Dt | 0 | D13 |
| GhWOX2b_At | 0 | A13 | GhWOX2b_Dt | 0 | D13 |
| GhWOX1_At | 1 | A12 | GhWOX10_Dt | 1 | D11 |

Note: GhWOX3a_Dt was located in scaffold, so it was not included in the gene type identification, and “-” is used to represent that. The identification was with A_t_ sub-genome or D_t_ sub-genome. 0, 1, 2, 3, 4 stand for singleton, dispersed, proximal, tandem, segmental.
