# Supplementary material for: Genome-wide analysis of WOX genes in upland cotton and their expression pattern under different stresses
Source: BMC Plant Biol. 2017 Jul 6;17:113. doi: 10.1186/s12870-017-1065-8 (PMC5501002; doi:10.1186/s12870-017-1065-8)
Supplement: Supplementary file 10 — Multiple sequence alignment of GhWOX5_At and GhWOX5_Dt. (PDF 47 kb) [file 12870_2017_1065_MOESM10_ESM.pdf]

```
Gh_WOX5_At     1 ATGGAAGAAGGGTTGTCGGGGTTTTGTATTAAAGGGAGTCATGTTCGTGGTAATAATGGG
Gh_WOX5_Dt     1 ATGGAAGAAGGGTTGTCGGGGTTTTGTATTAAAGGGAGTCATGTTCGTGGTAATAATGGG

Gh_WOX5_At    61 AACAACAATGGTGGTAGTGGAACTAAGTGTGGGAGATGGAACCCTACGACTGAACAGGTT
Gh_WOX5_Dt    61 AACAACAATGGTGGTAGTGGAACTAAGTGTGGGAGATGGAACCCTACGACTGAACAGGTT

Gh_WOX5_At   121 AAGGTGTTAACCGAACTGTTTAGGTCTGGACTCCGAACACCGAGTACTGATCAAATCCAG
Gh_WOX5_Dt   121 AAGGTGTTAACCGAACTGTTTAGGTCTGGACTCCGAACACCGAGTACTGATCAAATCCAG

Gh_WOX5_At   181 AAGATTTCTACACAGCTTAGCTTTTATGGGAAGATTGAAAGTAAGAATGTGTTTTATTGG
Gh_WOX5_Dt   181 AAGATTTCTACACAGCTTAGCTTTTATGGGAAGATTGAAAGTAAGAATGTGTTTTATTGG

Gh_WOX5_At   241 TTCCAGAATCATAAAGCTAGGGAAAGACAAAAGCGTAGAAAGGTCTCTATTGATGAAAAT
Gh_WOX5_Dt   241 TTCCAGAATCATAAAGCTAGGGAAAGACAAAAGCGTAGAAAGGTCTCTATTGATGAAAAT

                                                              A 6bp
Gh_WOX5_At   301 GATTTCATTTCTAGAGACAACATCTTCAATAACAACAACAA------GATTTCTTCTTCG
Gh_WOX5_Dt   301 GATTTCATTTCTAGAGATAACATCTTCAATAACAACAACAACAACAAGATTTCATCTTCG

Gh_WOX5_At   355 AAACGTAGGTTTCTCGTCTTCTTCTTCTTCTTCTTTT-AAACTATATTAGTAGTCAATCA
Gh_WOX5_Dt   361 AAACGTAGGTTTCTCGTCTTCTTCTTCTTCTTTTTTTCAAACTACATTAGTAGTCACTTA

Gh_WOX5_At   414 ACTATGGATTTTTTTTTGTTATACAATTACAAAAAGTTGTAGAATAATAATTTTCAATTT
Gh_WOX5_Dt   421 ACTGTGGGTTCTTTTT-GTTACCCAATTATGAAAAGTTATAAAATAATCGCTCTCAATTT

Gh_WOX5_At   474 GTTTTTTTTATATCACTCAACTATCTTTGATTTTTGG-TGTTTTTATTTTTATCATAGCT
Gh_WOX5_Dt   480 TTTT------TGTTACCTAACTATCTTAGATTTTTGGGTGTTTTCATTTTTACGATAGCC
                     B 6 bp

                                         C 29bp
Gh_WOX5_At   533 ATTTGATGATCAAAATAGATAAAATTAAATAATTGAGTGATTATTTTGTAATTTTTCATA
Gh_WOX5_Dt   534 ACTTGATGATCAAAATAGACAAAATTGAATAGTTAAGTGATCATTTTGTAACTTTTCATA

Gh_WOX5_At   593 ATTTAATA-----------------------------GCCTCTACTATAGTTTTCCCTTT
Gh_WOX5_Dt   594 GTTGAGTGATCAAAACAGAAAAAAAAAATAGTTGGGTGGCCTCTATTGTAGTTTACCTTTT

Gh_WOX5_At   624 TTGGGGGTCAGATCATGTTGTGTCGTGATTGTAAAATGATATTGTTATCTTTGTGTACTC
Gh_WOX5_Dt   654 TGGGGGGTCAGATGATGTTGTGTCATGATTGTAAGATGATATTGTCCCCTTTGTGTACTC

                     D 6 bp                                     E 14 bp
Gh_WOX5_At   684 ACTAACTCTTTAAGGTTTTCTCACTTGAACTTGCACTTCATAATCAAAT-----------
Gh_WOX5_Dt   714 ACTAA------AAGGTTTTTTCATCTGAAGTTGCACTTCATAATCAAATCCACGGCCTCC

Gh_WOX5_At   733 ---ATAAGAGTTTTATGTGGTAGTAAAAATGACTCGTATATGAAAAGGTATTGGGTTTGA
Gh_WOX5_Dt   768 CATATAGGCGTTTTACATGATAATGAAAATGACTTGTATATGACGAGGTATTGGATTTGA

                                                               F 29bp
Gh_WOX5_At   790 ATTTGGGGACGTACATTATTGGATAACAAATCGTTGAAGAA-------------------
Gh_WOX5_Dt   828 ATTTAGGAAAGTGCATTATCGGGTGACAAATTCTTGAAGAATTTGTGGAATTCAAAGAGT

Gh_WOX5_At   831 ----------CTTAAAAGTGGCATTATAATATATATTTCATTTGATTTGATGAGTTTTTT
Gh_WOX5_Dt   888 ACAATATGAACTTAAAAGTGATATTATAATAAACATTTCATTTGATTTGATGAGTTTTTT

                  G 5 bp
Gh_WOX5_At   881 TT-----AAAATTGGATCAACTTGAGTCATGTTTATAAGATGATATTGTTCTTTTTTATG
Gh_WOX5_Dt   948 TTTTTAAAAAATTGAGTCAAGT-ATGTCATGTCTATAAGATGATATTATCCTCTTT-ATG

Gh_WOX5_At   936 TACTTACTGATTCTTTAAGGTTTTCTCACTCGAAAATGCATGTTAGATTCAAATACAGGA
Gh_WOX5_Dt  1006 TACTCACTAATTCTTTAAGGTTTTCTCACTAAAAAATGTATCATAGATTCATATCCAGGG
```

H 3 bp

```
Gh_WOX5_At  996 ACTCCTCATATAAGAGTTCTATATAGTAGTGAAAATGACTAGAATATAAAGAGTTCTTAA
Gh_WOX5_Dt 1066 TCTCCCCATATAAGAGTTTTATGTGGTAGTGAAAATGAGTCGTATATGA-GAGGTCTTGA

Gh_WOX5_At 1056 GTTCGAACTTAAGGAAAATGCATTATTGGATGACAAATCTTT-AAGAGGACTTTAAAAAT
Gh_WOX5_Dt 1125 GTTCAAACCTAATAAAAGT--ATTATGAGATGACAAATCTTTTAAGAGAACTTAAAAAAA

Gh_WOX5_At 1115 AT---ATTATTATAACACATATTTCATTTGATTTAATGGGTTTTTTTT-CTTTAAATTTT
Gh_WOX5_Dt 1183 ATGGTATTATTATAACACATATTTCATTTGATTTAATGGGTTTTTTTTTCTTTAAATTTT

Gh_WOX5_At 1171 TGCAGGGTTTTTTGAGGTTAAGGAATATCAATCAGATCAAAGGGTTATTGAGACATTACA
Gh_WOX5_Dt 1243 TGCAGGGTTTTTCGAGGTTAAGGAATATCAATCAGATCAAAGGGTTATCGAGACATTACA

Gh_WOX5_At 1231 ACTATTTCCATTAAATTCCTTTGATGAAAATGAGCAAGAGAAGCTGAGATTCCATGCAAA
Gh_WOX5_Dt 1303 ACTATTTCCATTAAATTCCTTTGATGAAAATGAGCAAGAGAATCTGAGATTCCATGCAAA

Gh_WOX5_At 1291 CGAATGTAGGGAAACATCATCATTTCCATACACAATCAATAACCCAGAAATGGATCATCC
Gh_WOX5_Dt 1363 CGAATGTAGGGAAACATCATCATTTCCATACACAATCAATAACCCAGAAATGGATCATCC

Gh_WOX5_At 1351 ACCATTGGATCTTCGTTTAAGTTCCCTGTGA
Gh_WOX5_Dt 1423 ACCATTGGATCTTCGTTTAAGTTCCCTGTGA
```
