# Supplementary material for: Genome-wide analysis of WOX genes in upland cotton and their expression pattern under different stresses
Source: BMC Plant Biol. 2017 Jul 6;17:113. doi: 10.1186/s12870-017-1065-8 (PMC5501002; doi:10.1186/s12870-017-1065-8)
Supplement: Supplementary file 11 — Multiple sequence alignment of Gh_A11G2876 and Gh_WOX10_Dt. (PDF 51 kb) [file 12870_2017_1065_MOESM11_ESM.pdf]

```
                                            EXON 1
Gh_A11G2876     1 ATGGAGTGGGAGAATCAACATGGAGAAGAAGAGTACTTGCAGTACCAAATTCAGAATGGG
Gh_WOX10_Dt     1 ATGGAGCGGGAGAATCAACATGGAGAAGAAGAGTATTTGCAGTACCAAATTCAGAATGGG
                                            EXON 1
Gh_A11G2876    61 GTTTGCGGGAAAGTGATGAGTGATGAACAGGTAGAGGAACTGAAGAAACAGATCGTTGCC
Gh_WOX10_Dt    61 GTTTGCGGGAAAGTGATGAGTGATGAACAGGTAGAGGAACTGAAGAAACAGATCGTTGCC
                                            EXON 1
Gh_A11G2876   121 TATTCCGCTATTTCTGAACAGCTTGCTGAGTTGCACAAATCCATGTCTGCCCCCCACGAT
Gh_WOX10_Dt   121 TATTCAGCCATTTCTGAACAGCTTGCCGAGTTGCACAAATCCATGTCTGCCCGCCACGAT

Gh_A11G2876   181 TTTACTGGTTCTCTTCTCTTTTGTTTCTTTTGTTTCTTTCGACATGTTCCCCAGTTGAGT
Gh_WOX10_Dt   181 TTTACTGGTTCTCTTCTCTCTTGCTTCTTTTGTTTCTTTCGACATGTTCCCCAGTTGAGT

Gh_A11G2876   241 TCACCATTCGAATTCTTATTTGATCCGAATCGGCAATTTAGGTTTTACAGAAGCAAAAGT
Gh_WOX10_Dt   241 TCACCATTTGGATTCTTATTTGATCCAAATCGGCAATTTAGGTTTTACAGAAGCAAAAGT

Gh_A11G2876   301 GAAAAAAAAAAAGCTTTCATTGAAGGTTGCAGTCTTCTCTTTGAGAAGATGAAGATTTTA
Gh_WOX10_Dt   301 GAAAAAAAGA--GCTTTCATTGAAGGCTGCAGTTTTCTCTTTGAGAAGATGAAGATTTTA

Gh_A11G2876   361 AAGAAAGGCCACGTTTTTTTAGATGTTGGATTTTTAATTTATTCATTTATTTATTTTTAA
Gh_WOX10_Dt   359 AAGAAAGGCCACGTTTTTTTAGATGTTGGATTTTTAATTTATTCATTTATTTATTTTTAA

Gh_A11G2876   421 TATAATTTGGTTTTTTTTAAGAAGCAAATGTTGCTGGAAATTTTCATTAATTTCTAAAAC
Gh_WOX10_Dt   419 TATAATTTTGTTTTTTTT-AAGTAGCAAATGTTGTTGGGAATTTTCATTAATTTCTA----

Gh_A11G2876   481 CATTGTGTTCAAAAAATAAATATCAAGCAGTTTCTAGAATAAA-TTTTATATAAATATTA
Gh_WOX10_Dt   474 ------------AAAATAAACATCAAGCAGTTTCTAGAACAAAATTTTATATAAATATTA

Gh_A11G2876   540 TTTATAGTATTTTACAAACATTTTAAAAATATTGTTATGTTGGGTTTTTTAAATTATTTT
Gh_WOX10_Dt   522 TTTATAGTATTTTACAAACATTTTAAAAATATTGTTATGTTGGGTTTTTTAAAATATTTT

Gh_A11G2876   600 ATATGTAGATGGTATATTTATATTCTTTTAATTAATTTAAAATATATATATTGTATTATA
Gh_WOX10_Dt   582 ATATGTAGATGGTC----------------------------------------------

Gh_A11G2876   660 TCTAAAACTGGTCATGGGATTGTTTATCTAAT-TTGAAAACTCGTTTGAAAAGTGTAAGG
Gh_WOX10_Dt   596 ----------------GGATTGTTTATCCAATCTTAAAAATTTGTTTGAAAAGTGCAAGG

Gh_A11G2876   719 ATTTAGTTAGAAATATAAACTCAAAAATTAAGATGTATAAAAAAAAAGT-CCGTTTTTTA
Gh_WOX10_Dt   640 GTTTAGTTAAAAATATAGATT-AAAAATGGGGTTGGATAAAAAATAACTTCCGTTTTTTA

Gh_A11G2876   778 AATGGGTTGGGTATTAAGTGGTTTTTTTTTTGTTTTGGCTTGGCTTAAATCAGTTGTTTT
Gh_WOX10_Dt   699 AATGAGTTGGGCCTCAGGTGGTGTTTTTTG-GTTTCAGCTTGGCTTAAATCAGTTGTTTT

Gh_A11G2876   838 GTT-----------ATCATTTTGTTATTATATTATTGTTATTGTTTTGATATTATATAAC
Gh_WOX10_Dt   758 GTTGTTGTTTTGCTATCATTTCGTTATTATATTGTTGTTATTATTTTGATATTATATAAC

Gh_A11G2876   887 TCTTGTTTTATTATTAATTTTGTTATTATTTTAGACATTTTTGCTTGATAAGATGCAACT
Gh_WOX10_Dt   818 TCTTGTTTTATTATTAACTTTGTTATTATTTTAGACATTTTTGCTTGATAAGATGTAATT

Gh_A11G2876   947 ATTTTAGTGTTATTCAAATTAATTTAATAAGAATAGGTCGGGATGGATTTGAGTTTAGTA
Gh_WOX10_Dt   878 ATTTTAGTATTATTTAAATTAATTTAATACGAGTGGGTCTGAATGGATTTGGGTTTAGTA

Gh_A11G2876  1007 TTTTTAAT--AGGCTAAGCTTAGATAGAATTTTAGATTCATTTTCTGAGTTGAATCTGAA
Gh_WOX10_Dt   938 TTTTTAATTTGGGTTGAGCTTAGGTAGAATTTTAGATTTATTTTTTGAGTCTGATCTTAA
```

```
Gh_A11G2876 1065 ATTAAAATCTTGCATCTGACTGACCCATGATAAGAT------------------------
Gh_WOX10_Dt  998 CTTAAAATCTTGTATC-AACCGACCCATAATCAGATTTAATTCTACTTTATAGAAATAAA

Gh_A11G2876 1101 ---------------------------------------------------------AAT
Gh_WOX10_Dt 1057 TTGTGAGAATCGTAAAATTGTATTTCCTTTTTGTATGACTAGTCTATCATATTGCTTAAT

Gh_A11G2876 1104 GCTTACCAAATAAATAACAGATTTTGCTTCCGATTGTGTTACTCGTACTGCATCTCGTCC
Gh_WOX10_Dt 1117 GCTTACCAAATAAATAACAGGTTTTGTTTCCGATTTTGTTCCTCGTACTGCATCTGGTCT

Gh_A11G2876 1164 AAGTACCATAGAGTTAAAAGCTTAGAGAAAATCGTTGTCTGGCTGCCTTAGTCCTATCAT
Gh_WOX10_Dt 1177 AAGTACCATAGAGTTAAAAGCTTAGAGAAAATCGTTGGCTGGCTGCCTTAGTCCTATCAT
                                          EXON 2
Gh_A11G2876 1224 CAATTGCTGTTTTGGTTTCAGGCATAAGGTTGGGAAATCTATACAGTGATCCAATATCAG
Gh_WOX10_Dt 1237 CAATTGCTGTTTTGGTTTCAGGCATAAGGTTGGGAAATCTATACTGTGATCCAATATCAG
                                          EXON 2
Gh_A11G2876 1284 CTTCTGTCGGGCAGAAGATCACTGCTAGACAGCGATGGACTCCAACGCCATTACAACTTC
Gh_WOX10_Dt 1297 CCTCTTTTGGGCACAAGATCACTCCTAGACAGCGATGGACTCCAACGCCATTACAACTTC
                                          EXON 2
Gh_A11G2876 1344 AAATTCTTGAGAATATATATGAGCAAGGAACCGGGACACCAAGCAAGCAGAAAATCAAAG
Gh_WOX10_Dt 1357 AAATTCTAGAGAATATATATGAGCAAGGAACCGGGACACCAAGCAAGCAGAAAATCAAAG
                                          EXON 2
Gh_A11G2876 1404 AGATAGCATCTGAACTAGCCCAACATGGTTGA----------------------------
Gh_WOX10_Dt 1417 AGATAGCATCTGAACTAGCACAACATGGTCAAATTTCTGAAACTAATGTATATAATTGGT
                                          EXON 2
Gh_A11G2876
Gh_WOX10_Dt 1477 TTCAGAATCGTCGTGCTCGTTCAAAAAGAAAATTTCAGGTCTCTACTGGTTCAGCCAATG
                                          EXON 2
Gh_A11G2876      ------------------------------------------------------------
Gh_WOX10_Dt 1537 CTGAACCTGAACCAGATGTGAGCACAAAAGAGAAAAAGACCAAACCAGTAGGTCTGGACT
                                          EXON 2
Gh_A11G2876      ------------------------------------------------------------
Gh_WOX10_Dt 1597 TCATTGACAGCTTCTCACAAGGGGTTGAAAGCTTCTATTTCCAAGGTTCTGAATCAGGTA

Gh_A11G2876      ------------------------------------------------------------
Gh_WOX10_Dt 1657 CTAAAATGTAGCATTTGGTCTTGTGTCGAAATCATAACTTTCCTTTTTTTTAAAAAGTTC
                                          EXON3
Gh_A11G2876      ------------------------------------------------------------
Gh_WOX10_Dt 1717 TTTTTGCAGGGATTGATCAGTTGATGGGTAAAGAGGAATCTTCACGAGGCTATGATCCTT
                                          EXON3
Gh_A11G2876      --------------------------------------
Gh_WOX10_Dt 1777 ACAATAATTTAGTTGAACAATTTGGGTTATTAGGATGA
```
