# Supplementary material for: Genome-wide analysis of WOX genes in upland cotton and their expression pattern under different stresses
Source: BMC Plant Biol. 2017 Jul 6;17:113. doi: 10.1186/s12870-017-1065-8 (PMC5501002; doi:10.1186/s12870-017-1065-8)
Supplement: Supplementary file 13 — The primers used in present study for qPCR. (DOCX 16 kb) [file 12870_2017_1065_MOESM13_ESM.docx]

| Gene locus name | Primer direction | Sequence (5’-3’) | Application |
| --- | --- | --- | --- |
| GhWOX1_At | Forward | AGCAGCAGTGTCAGAATGTAGAG | qPCR(Candidate genes) |
|  | Reverse | AGAACAGGTGATAGGCATGAGTAA |  |
| GhWOX10_Dt | Forward | AACATGGAGAAGAAGAGTATTTGCA |  |
|  | Reverse | CAAGCTGTTCAGAAATGGCTGAATA |  |
| GhWOX13a_At/Dt | Forward | GGCTCGGTCTAAAAGGAAGCAACT |  |
|  | Reverse | GGTTCTGCCCAGAGAGAAGATTCTC |  |
| GhWOX13b_At/Dt | Forward | GTGAAGGTGATGACAGATGAGCAG |  |
|  | Reverse | ATACAGATTTCCCAGTCTCCCACCT |  |
| GhWOX4a_At/Dt | Forward | AATGGCCCCTCTGAAGACAACAAC |  |
|  | Reverse | CCCCGATACAACATCTCCAAAATC |  |
| Ghhistone3 | Forward | TCAAGACTGATTTGCGTTTCCA | qPCR (Internal control) |
|  | Reverse | GCGCAAAGGTTGGTGTCTTC |  |
